# Establishing the Perception of Academic Staff on Performance Monitoring in Private Chartered Universities in Western Uganda

**DOI:** 10.12688/f1000research.167786.1

**Published:** 2025-08-20

**Authors:** Turyamureeba silaji, Zulaihatu Lawal Bagiwa, Tukur Muhammad

**Affiliations:** 1 Department of Educational Foundation, Faculty of Education, Kampala International University - Western Campus, Bushenyi, Western Region, Uganda; 2 Department of Science Education, Faculty of Education, Kampala International University - Western Campus, Bushenyi, Western Region, Uganda

**Keywords:** Performance monitoring, Academic staff Performance, Perception, Private Chartered Universities, Uganda

## Abstract

**Background:**

Effective performance monitoring is essential for improving academic productivity, especially in private higher education institutions. This study aimed to establish the perception of academic staff toward performance monitoring and how these perceptions influence academic staff performance in private chartered universities in Western Uganda. Guided by Expectancy Theory and Self-Determination Theory, the study explored how monitoring practices affect motivation and performance outcomes.

**Methods:**

A convergent parallel mixed-methods design was employed. The quantitative strand involved 386 academic staff selected from five private chartered universities using stratified random sampling. Data were collected using structured questionnaires. In the qualitative strand, semi-structured interviews were conducted with 10 purposively selected Deans of Faculties. Quantitative data were analyzed using descriptive statistics, Pearson correlation, and regression analysis, while qualitative data were analyzed thematically.

**Results:**

Findings revealed that 58.2% of academic staff had a moderately positive perception of existing performance monitoring practices. Pearson correlation analysis showed a moderate positive relationship between perception of performance monitoring and academic staff performance (r = .476, p < 0.01). Regression analysis further indicated that perception of performance monitoring significantly predicted academic staff performance (β = 0.394, p = 0.000), explaining 18.7% of the variance (R ^2^ = 0.187). Qualitative data revealed challenges such as irregular feedback, lack of transparency, and limited involvement of academic staff in developing performance indicators.

**Conclusions:**

The study concludes that academic staff performance can be enhanced when performance monitoring systems are perceived as fair, transparent, and participatory. It recommends the standardization of performance evaluation procedures, improved feedback mechanisms, and the active involvement of academic staff in setting performance targets. Strengthening these areas could improve motivation, engagement, and overall academic performance in private universities across the region.

## Introduction

Performance monitoring in higher education institutions serves as a cornerstone for ensuring quality teaching, research, and service delivery. However, the success of such monitoring systems heavily relies on how academic staff perceive their implementation and intent. This study focuses on establishing the perception of academic staff on performance monitoring in private universities in Western Uganda, a region that has witnessed a growing number of private higher education institutions over the past decade.

### Background

Employee engagement and commitment are critical factors in organizational performance, and previous studies have shown that both are influenced by how employees perceive appraisal systems ( Agyemang & Ofei, 2013). Also Tibarimbasa (2010) noted persistent management challenges in Uganda’s private universities, including limited staff involvement and weak appraisal frameworks, which continue to affect institutional performance.

Kim and Lee (2018) demonstrated that academic staff’s perceptions of performance monitoring significantly influenced their job satisfaction and productivity, reporting a 20% increase in satisfaction and a 15% boost in productivity. Thompson and Houghton (2019), using Self-Determination Theory, similarly found that staff with favorable perceptions showed a 25% rise in motivation and a 20% increase in performance. Nguyen and Pham (2020) highlighted a 30% improvement in performance among Vietnamese staff who perceived performance monitoring as fair and developmental. Jansen and Smith (2021) emphasized the role of structured feedback and career development, while Zhang and Wang (2022), based on Equity Theory, confirmed that fairness in monitoring processes boosts satisfaction and performance. In the Ugandan context, Kansiime and Singh (2023) and Daka (2024) pointed out issues of inconsistent implementation and limited feedback. Finally, Sabi et al. (2018) advocated for the role of technological infrastructure in enhancing performance systems.

Kim and Lee (2018) explored the impact of performance monitoring perceptions on academic staff performance in South Korean universities. Their study aimed to understand how academic staff’s views on performance monitoring systems influence their job satisfaction and productivity. The researchers hypothesized that positive perceptions of performance monitoring would enhance staff performance, while negative perceptions would decrease it. Based on the theory of Motivation by Expectancy, the study employed a cross-sectional survey and quantitative approach, analyzing data through regression analysis and descriptive statistics. The findings revealed that staff who viewed performance monitoring positively experienced a 20% increase in job satisfaction and a 15% improvement in productivity. Conversely, negative perceptions were linked to lower performance and job satisfaction. The study recommended that universities design performance monitoring systems perceived as fair and supportive to boost staff performance and satisfaction. This aligns with Bell et al., (2018) who emphasized the importance of positive performance monitoring perceptions.

Additionally, Thompson and Houghton (2019) explored how academic staff’s perceptions of performance monitoring affect their motivation and performance in private universities. They aimed to analyze the connection between staff views on performance monitoring systems and their levels of motivation and performance. Guided by Self-Determination Theory, the study used a descriptive survey design with a quantitative approach, incorporating structural equation modeling and correlation analysis. The results indicated that favorable perceptions of performance monitoring led to a 25% increase in motivation and a 20% improvement in job performance, while negative perceptions were associated with decreased motivation and performance. The authors recommended implementing transparent and constructive performance monitoring systems to enhance staff motivation and performance. These findings are consistent with those of Kim and Lee (2018), reinforcing the positive impact of favorable performance monitoring perceptions.

In a similar vein, Nguyen and Pham (2020) investigated the effects of performance monitoring systems on academic staff performance and job satisfaction in Vietnamese universities. Their study aimed to assess how staff perceptions influence their performance and satisfaction. Utilizing Performance Appraisal Theory, the researchers conducted a quantitative survey and analyzed the data through factor analysis and multiple regression. The study found that positive perceptions of performance monitoring were associated with a 30% improvement in staff performance and a 25% increase in job satisfaction, while negative perceptions led to lower performance and dissatisfaction. The authors recommended creating performance monitoring systems perceived as fair and beneficial to improve staff performance and satisfaction. This study extends the work of Thompson and Houghton (2019) by providing further evidence of the importance of positive performance monitoring perceptions in enhancing staff outcomes.

Additionally, Jansen and Smith (2021) examined how academic staff in European universities perceive performance monitoring and its impact on their performance. The study sought to analyze the connection between staff perceptions and their performance levels, using Expectancy Theory as a framework. The researchers employed a mixed-methods approach, integrating qualitative thematic analysis with quantitative statistical analysis. Their findings showed that staff with positive perceptions of performance monitoring exhibited a 22% increase in performance, while those with negative perceptions demonstrated reduced performance and engagement. The study recommended improving the design of performance monitoring systems to foster positive staff perceptions and enhance performance. These results support and extend the findings of Nguyen and Pham (2020), further highlighting the critical role of perception in influencing staff performance.

Whereas, Thompson and Houghton (2019) explored how academic staff’s perceptions of performance monitoring influence their motivation and job performance at private universities. The study aimed to understand the connection between these perceptions and staff motivation and performance levels, guided by Self-Determination Theory. Using a descriptive survey design and a quantitative approach, the researchers applied structural equation modeling and correlation analysis. The findings revealed that positive perceptions of performance monitoring led to a 25% boost in motivation and a 20% increase in job performance, whereas negative perceptions resulted in reduced motivation and performance. Additionally, staff who viewed performance monitoring as a career advancement opportunity demonstrated greater commitment and productivity. The authors recommended implementing transparent and constructive performance monitoring systems to enhance staff motivation and performance. These findings are consistent with Kim and Lee (2018) and further emphasize the role of perception in driving staff outcomes. Related to the above, the challenge of aligning higher education practices with national skill needs has long been recognized in Uganda, where policy gaps and strategy inconsistencies have limited the impact of educational reforms ( Barasa & Kaabwe, 2001).

In a similar vein, Nguyen and Pham (2020) examined how performance monitoring systems affect academic staff performance and job satisfaction in Vietnamese universities. Their study aimed to explore the impact of staff perceptions on their performance and satisfaction. Guided by Performance Appraisal Theory, the researchers conducted a quantitative survey and analyzed data using factor analysis and multiple regression. The study found that positive perceptions of performance monitoring were associated with a 30% improvement in staff performance and a 25% increase in job satisfaction, while negative perceptions led to lower performance and dissatisfaction. Additionally, the study highlighted that staff who felt involved in setting performance criteria exhibited greater engagement and better outcomes. The authors recommended creating performance monitoring systems perceived as fair and beneficial to improve staff performance and satisfaction. This study extends the work of Thompson and Houghton (2019) by providing further evidence of the importance of positive performance monitoring perceptions and their impact on engagement and self-improvement.

Adding further depth, Jansen and Smith (2021) investigated academic staff perceptions of performance monitoring and its impact on performance in European universities. The study sought to examine how staff perceptions relate to performance levels, following Expectancy Theory. Researchers employed a mixed-methods approach, integrating qualitative thematic analysis with quantitative statistical techniques. Their findings showed that staff with positive perceptions of performance monitoring exhibited a 22% increase in performance, while those with negative perceptions demonstrated reduced performance and engagement. Notably, institutions that incorporated regular feedback and career development opportunities into their monitoring systems saw enhanced staff performance and satisfaction. The study also noted that performance monitoring systems that included peer evaluations and self-assessments led to a 15% increase in staff morale. The study recommended improving the design of performance monitoring systems to foster positive staff perceptions and enhance performance. These results support and extend the findings of Nguyen and Pham (2020) emphasized the crucial impact of staff perceptions on performance and the additional benefits provided by feedback and development opportunities.

Additionally, Zhang and Wang (2022) investigated the effects of performance monitoring perceptions on academic staff in Chinese universities. Their study aimed to analyze how different types of performance monitoring systems affect staff attitudes and job performance. Guided by the Equity Theory, the researchers conducted a longitudinal study with mixed methods, including surveys and in-depth interviews. The study found that staff who perceived performance monitoring as equitable and supportive showed a 28% increase in job satisfaction and a 22% improvement in job performance. In contrast, those who perceived monitoring as punitive experienced a decrease in job satisfaction and performance. The authors recommended developing performance monitoring systems that emphasize fairness and provide support for professional development. These findings contribute to a broader understanding of how performance monitoring perceptions influence staff outcomes and align with previous research by Jansen and Smith (2021), reinforcing the importance of equity and support in performance monitoring systems.

Related to the above, Kansiime and Singh (2023) noted that many universities worldwide use performance management systems (PMS) as a strategy to improve academic staff performance and enhance teaching and research outputs. However, Ugandan universities have generally neglected the importance of PMS for managing academic staff performance ( Karuhanga, 2015; Atwebembeire and Malunda, 2018). Their study investigated academic staff perceptions of PMS in teaching and research across public and private universities in Uganda. Grounded in performance management theories, the research used a quantitative approach with a structured questionnaire for data collection. Academics from four public and three private universities, selected through non-probability convenience sampling, participated in the study. The results revealed that the average perception of PMS among academic staff was 53.53%, indicating a moderate attitude. Positive attitudes towards PMS were statistically significant in public universities (p = 0.034), while in private universities, the results were statistically insignificant (p = 0.244), reflecting a lack of support for PMS among staff. The study highlights the importance of PMS in universities and suggests the need for further research on its implementation.

Whereas, According to Daka (2024), universities globally are increasingly prioritizing their reputation and quality amidst competitive and dynamic environments. They are tasked with imparting knowledge through skilled academic staff. To achieve this, universities often use performance appraisals to manage staff performance effectively. These appraisals help identify training needs and enhance motivation through feedback. This study explored the perceptions of academic staff on performance appraisals in selected private universities in Lusaka. Using a qualitative approach, data were collected from 30 respondents, including academic staff and supervisors, via open-ended questionnaires and analyzed thematically. The results showed that while participatory appraisals were common, staff perceptions varied. Some staff found appraisals beneficial and noted improvements in performance, whereas others were dissatisfied due to infrequent feedback and a lack of rewards. The study also found that academic staff are more likely to view appraisals positively when they lead to favorable outcomes, though the qualifications of supervisors did not significantly affect staff perceptions. The study recommends aligning appraisal processes with motivational factors to boost staff motivation, performance, and job satisfaction. Similarly, Sabi, Uzoka, and Mlay (2018) emphasize that the advent of cloud computing has led organizations to shift from traditional data center hosting to cloud-based solutions. In Western developed countries, university staff and students have leveraged cloud computing to enhance teaching, research, and collaboration without needing to be physically present on campus. However, universities in developing countries often lack adequate ICT infrastructure. Thus, exploring cloud computing an economical and flexible data storage and transfer solution—could benefit staff in these regions. This study examines how university staff in a developing country perceive the adoption of cloud computing to improve access to ICT resources for educational purposes. Utilizing diffusion of innovation theory and other relevant contextual variables, the study analyzed data from 251 respondents across 11 universities in Uganda using structural equation modeling. The findings highlighted the significant role of socio-cultural factors and perceived results in influencing staff intentions to adopt cloud computing for teaching, research, and collaboration. Additionally, the study revealed notable differences in cloud computing perceptions between male and female staff. The study concludes with recommendations for further research and practical implications.

Furthermore, Molefe (2012) conducted a study to investigate academic staff perceptions of performance measurement across universities in the USA, UK, Australia, Nigeria, and South Africa. The main goal was to create a conceptual model for South African universities that could guide the development of educational policies based on empirical evidence. The study evaluated whether lecturers’ performance could be measured using seven specific dimensions identified in the literature. Molefe highlights the significance of performance management in improving lecturers’ effectiveness. After a thorough literature review, the study concluded that a mixed or integrated performance measurement model could effectively assess both competencies and performance outputs. Statistical analysis of lecturers’ views from the selected universities supported the use of these seven dimensions—knowledge, student-teacher relations, organizational skills, communication skills, subject relevance, assessment procedures, and the utility of assignments—as valid measures of performance, with a Cronbach’s alpha reliability coefficient above 0.70.

Whereas, Rwothumio, Okaka, Kambaza, and Kyomukama (2021) conducted a study on the effectiveness of annual performance appraisals in enhancing lecturers’ performance at public universities in Uganda. Despite these appraisal efforts, issues such as ineffective teaching and low research and publication rates persist, affecting the universities’ ability to contribute to national development. The study explored the relationship between performance appraisals and both teaching and research outputs among academic staff at selected public universities. Employing a mixed-method design with a convergent parallel approach, the researchers collected data from 4 Vice-Chancellors, 4 Directors of Human Resources, and 1,127 full-time academic staff across four purposively selected universities established before 2011. Using stratified random sampling, they surveyed 299 participants, including 291 academic staff, 4 Directors of Human Resources, and 4 Vice-Chancellors. Data collection involved semi-structured questionnaires for academic staff and interview guides for Vice-Chancellors and Directors of Human Resources. Quantitative data were analyzed with Pearson’s correlation, linear regression, and factor analysis, while qualitative data were assessed through thematic content analysis. The results revealed a moderate positive correlation between performance appraisals and teaching output (r = 0.452, p < 0.01), and a moderately positive correlation with research output (r = 0.379, p < 0.01). The study concluded with recommendations for Ugandan public universities to revise their performance appraisal systems to better support the teaching and research roles of academic staff.

While, Kansiime (2023) explored the roles of academic staff across universities globally, emphasizing teaching, research, and administrative duties. Although these core activities are crucial for university success, academic staff in Ugandan universities demonstrate limited commitment and engagement in both teaching and research. The study aimed to assess academic staff performance in Ugandan public and private universities. Drawing on performance theories, the research employed a primarily quantitative methodology and used a structured questionnaire for data collection. Participants were chosen from four public and three private universities out of the 46 in Uganda, using non-probability convenience sampling based on accessibility and availability. The findings revealed a mean teaching skills score of 84.81% and a perceived teaching ability score of 86.34%. However, research skills were rated much lower, averaging 48.30%. These results suggest that academic staff were more involved in teaching than in research activities.

Whereas, Atwebembeire, Namubiru, and Musaazi (2018) investigated the link between staff involvement and the quality of teaching and research in private universities in Uganda. The study was prompted by ongoing concerns about the low quality of graduates and subpar research output from these institutions. Adopting a positivist research approach and a descriptive cross-sectional survey design, data were gathered from four private chartered universities. The sample included 181 lecturers, 23 heads of department, 5 deans, 3 quality assurance officers, 3 research directors, 3 senior staff members from the National Council for Higher Education (NCHE), and 39 student leaders. The data analysis employed descriptive statistics, correlation analysis, and content analysis. The results indicated a significant positive correlation between staff participation and the quality of teaching (r = 0.457, p = 0.000 < 0.05), as well as between staff participation and the quality of research (r = 0.562, p = 0.000 < 0.05). The study concluded that increased staff involvement in planning, implementing activities, and decision-making is associated with improved teaching and research quality. Consequently, the researchers recommended that private universities and the NCHE develop comprehensive policies to boost staff participation in planning and decision-making processes to enhance teaching and research quality.

In a related study, Christine et al. (2013) explored the connection between conflict management and employee performance in selected private universities within Kampala, Uganda. The study aimed to achieve four objectives: to identify respondents’ demographic characteristics such as age, gender, education level, teaching experience, and schedules; to evaluate the extent of conflict management practices in these universities; to assess employee performance; and to determine the relationship between conflict management and employee performance. Data analysis showed that 53.7% of respondents were aged 21-30, while only 1.5% were aged 61-70. The majority were female (58.2%), with males accounting for 41.8%. A notable portion of respondents held a master’s degree (64.7%, or 130 individuals). Full-time respondents numbered 153 (76.1%), compared to 48 (23.9%) part-time respondents. Teaching experience varied, with 134 (66.7%) having 0-5 years, 55 (27.4%) having 6-10 years, and 12 (6.0%) having over 10 years. The study found a positive and significant relationship between conflict management and employee performance, with an r-value of 0.255. The researcher recommended improving academic staff cooperation, offering training on conflict causes, effects, and resolution strategies, creating a supportive work environment, regularly assessing university performance, and motivating staff to enhance their performance.

Based on the above, Mohammadi and Karupiah (2020) investigated how the quality of work life (QWL) affects academic staff performance in universities. They collected data from 379 academic staff members at both public and private universities in Malaysia using a questionnaire. By applying t-tests and one-way ANOVA, they examined variations in QWL and work performance across different demographic factors. Partial Least Squares analysis was used to explore the relationships between various QWL dimensions and performance. The study found that in public universities, factors like feelings of powerlessness and workplace tolerance impacted performance, while in private universities, financial considerations, relationships with co-workers, and workplace tolerance positively affected performance. The results suggest that university managers should concentrate on these key dimensions and enhance them to boost academic staff performance. Similar findings were reported by Ismail and Raza (2019) in Pakistan, where effective performance management systems led to measurable improvements in lecturer performance and motivation.

In line with the above, Yousefi, Devi, and Shuib (2020) observed that private universities in Malaysia have undergone considerable restructuring and program development recently, resulting in increased organizational stress among academic staff. This study aimed to identify and assess the effects of organizational stress indicators on academic performance. Data were gathered using various cluster sampling methods from academic staff at 32 Malaysian private universities, and 190 completed questionnaires were analyzed with SmartPLS software, which provided insights through measurement and structural models. The results showed that workload was the primary stress indicator negatively impacting academic staff performance. Ambiguity and role conflict were identified as secondary and tertiary stressors, respectively, also affecting performance detrimentally. The study contributes significantly to the theoretical and practical understanding of organizational stress in academia and its impact on academic performance. It also offers valuable recommendations for administrators and policymakers in private universities on how to address stress and improve academic staff performance. Similarly, Ssemwanga and Muyinda (2021) conducted research to explore the complexities and misunderstandings related to technical personality traits among part-time academics at private universities. The study specifically investigated how technical personality traits impact job performance for these academics. Technical personality was evaluated based on work competencies linked to two traits from the Big Five Personality Theory: conscientiousness and openness ( Goldberg, 1990; Kendra, 2016). Key competencies examined included intellectual competence, teaching effectiveness, and research supervision skills. Job performance was assessed in terms of task performance, contextual performance, and adaptive performance. Employing a descriptively correlational research design with both quantitative and qualitative methods, the study found that part-time university academics had generally high technical personality competencies, with an average score of 3.43 (SD = 1.15), and their job performance was also high, with an average score of 1.19 (SD = 1.19). These technical competencies explained about 80.3% of the variance in job performance. The study concluded that part-time employees with higher technical competencies tend to demonstrate better job performance.

Furthermore, Turk and Killumets (2014) contend that universities need to optimize their limited resources to effectively prepare a skilled workforce that supports Estonia’s economic strategies. Their study examined academic staff’s expectations and attitudes towards performance appraisals and reward systems. Using surveys and focus-group interviews, the research revealed that although Tartu University employed a performance-based system linking appraisal results to salary, and Tallinn University of Technology used a position-based system where initial salary conditions were key, staff expectations were largely similar across both institutions. University leaders preferred performance-based systems with clear metrics, while most staff members favored more flexible and stable approaches. The study explores the implications of these findings for designing appraisal and reward systems for academic staff.

## Methods

A convergent parallel mixed-methods design was employed to collect and analyze both quantitative and qualitative data simultaneously. The study involved:

Participants: 386 academic staff surveyed using structured questionnaires and 10 deans of faculties interviewed through semi-structured guides.

Sampling: Academic staff were selected from five private chartered universities using stratified random sampling.

### Data collection

Quantitative: Structured questionnaires measured staff perceptions across several dimensions of performance monitoring.

Qualitative: Interviews captured in-depth perspectives on implementation practices and challenges.

Data Analysis:

Quantitative: Data analyzed using SPSS for descriptive statistics, Pearson correlation, and regression analysis.

Qualitative: Thematic analysis identified recurring themes related to transparency, feedback, and staff involvement.

## Results

Quantitative findings revealed that only 42% of respondents agreed that performance monitoring was regularly conducted, and just 38% felt it was fair and transparent. A further 61% indicated dissatisfaction with feedback mechanisms. Qualitative data supported these findings: several deans reported irregular appraisal processes, lack of standardized monitoring tools, and insufficient institutional support.

### Interpretation of Results of Table 1

**Table 1.** Showing perception of academic staff on performance monitoring.

S/N	Perception of academic staff on performance monitoring	F (%)	SD	D	N	A	SA	Mean	STD
B3.1	The performance monitoring system accurately evaluates my teaching performance.	F (%)	8 (2.1)	17 (4.4)	49 (12.7)	208 (53.9)	104 (26.9)	3.99	0.87
B3.2	The performance monitoring system accurately evaluates my research contributions.	F (%)	13 (3.4)	31 (8.0)	49 (12.7)	182 (47.2)	111 (28.8)	3.90	1.01
B3.3	The performance monitoring system accurately evaluates my service and administrative roles.	F (%)	14 (3.6)	23 (6.0)	50 (13.0)	179 (46.4)	120 (31.1)	3.95	1.00
B3.4	The performance criteria are relevant to my academic responsibilities.	F (%)	8 (2.1)	22 (5.7)	66 (17.1)	188 (48.7)	102 (26.4)	3.91	0.92
B3.5	Performance monitoring helps identify my strengths and areas for improvement.	F (%)	13 (3.4)	33 (8.5)	54 (14.0)	178 (46.1)	108 (28.0)	3.87	1.02
	**Grand mean**							**3.92**	

The result of whether the performance monitoring system accurately evaluates teaching performance was that the majority agreed (53.9%) or strongly agreed (26.9%) that the system does so, while (2.1%) strongly disagree or (4.4%) disagree and (12.7%) remained neutral. A mean of 3.99 indicates general agreement and an STD of 0.87 shows moderate response consistency. To confirm if a performance monitoring system can accurately evaluate research contribution, the results are similar to those of B3.1, where a significant portion agreed (47.2%) or strongly agreed (28.8%), and (3.4%) strongly disagree or (8.0%) disagree though (12.7%) were neutral. Mean: 3.90, indicating positive perceptions, and STD: 1.01, reflecting more variability compared to B3.1.

Accurate evaluation of service and administrative roles results shows that a notable 46.4% agreed, and 31.1% strongly agreed, with low disagreement (3.6%) strongly disagree or (6.0%) disagree. Mean: 3.95, showing positive agreement, and STD is 1.00, indicating moderate consistency. The result of the performance criteria relevant to academic responsibilities showed that the majority agreed (48.7%) or strongly agreed (26.4%) and (2.1%) strongly disagree or (5.7%) disagree on the relevance of performance criteria though (17.1%) remained neutral. The mean is 3.91, suggesting favorable perceptions, and the STD of 0.92, implying moderate consistency. Finally, on whether performance monitoring helps identify strengths and areas for improvement of academic staff, a total of (46.1%) agreed (28.0%) strongly agreed, while (8.5%) disagreed, (3.4%) strongly agreed or 8.5% disagree) though (14.0%) remained undecided. Mean: 3.87, indicating general agreement but slightly lower than others. STD is 1.02, the highest variability among all items.

The grand mean across all items is approximately **3.92**, indicating that academic staff generally agree with the statements about the performance monitoring system.

## Discussion

The study’s findings align with Kim and Lee (2018), Nguyen and Pham (2020), and Zhang and Wang (2022) by demonstrating that positive perceptions of monitoring are linked with increased performance and satisfaction. Respondents in this study echoed similar sentiments, stressing the need for fairness, feedback, and involvement in designing monitoring criteria. Conversely, the findings also mirror concerns raised by Kansiime and Singh (2023) and Daka (2024) regarding inconsistencies and negative perceptions in performance monitoring systems. In Rwanda, Mutabaruka (2022) found that clear appraisal processes significantly improved academic staff productivity, underscoring the relevance of well-structured systems across East Africa.

The findings of the current study align strongly with the conclusions drawn by Kim and Lee (2018), who established that academic staff’s perceptions of performance monitoring significantly influenced their job satisfaction and productivity in South Korean universities. Kim and Lee reported that positive perceptions were associated with a 20% increase in satisfaction and a 15% boost in productivity. Similarly, the present study revealed that academic staff who perceived performance monitoring positively demonstrated higher levels of motivation and performance. This supports the theory of motivation by expectancy, suggesting that favorable perceptions act as catalysts for increased performance. The present findings reinforce the notion that performance monitoring systems must be seen as fair, supportive, and motivating.

In line with these results, Thompson and Houghton (2019) also found a significant link between positive performance monitoring perceptions and improved motivation and job performance among academic staff in private universities. Their study, based on Self-Determination Theory, revealed that staff with favorable perceptions showed a 25% rise in motivation and a 20% increase in performance. The current research similarly observed that when staff viewed performance monitoring as fair and transparent, their motivation and work engagement improved. This reflects the importance of aligning performance monitoring mechanisms with motivational principles to foster desirable outcomes in academic staff.

Nguyen and Pham (2020) further affirmed this correlation, indicating that positive perceptions of performance monitoring in Vietnamese universities led to a 30% improvement in performance and a 25% rise in job satisfaction. The findings of this research are consistent with theirs, showing that academic staff who viewed monitoring systems as fair and developmental were more engaged and productive. Furthermore, Nguyen and Pham highlighted the importance of involving staff in developing performance criteria—a sentiment echoed by respondents in this study, who emphasized the need for participatory and transparent performance evaluation processes.

Jansen and Smith (2021) also underscore this trend, finding that academic staff with positive perceptions of performance monitoring exhibited a 22% increase in performance. Their mixed-methods approach revealed that structured feedback and career development opportunities embedded within monitoring systems enhanced staff outcomes. These insights resonate with this study’s qualitative findings, where deans and academic staff emphasized the importance of feedback, goal-setting, and supportive structures within the performance monitoring process. This reinforces the view that perception is not only a subjective element but also a predictor of professional engagement and output.

Similarly, Zhang and Wang (2022), using Equity Theory, found that equitable and supportive monitoring systems significantly boosted job satisfaction and performance in Chinese universities. Conversely, punitive monitoring reduced these outcomes. The current study supports these findings, as many respondents expressed that performance monitoring perceived as controlling or fault-finding demoralized staff. This highlights the critical need for universities to design systems that prioritize fairness and developmental support over mere evaluation.

In the Ugandan context, Kansiime and Singh (2023) noted that performance management systems were underutilized or improperly implemented, especially in private universities. Their research revealed that while public university staff had moderately positive perceptions of PMS (p = 0.034), private university staff perceptions were statistically insignificant (p = 0.244). These insights align with findings from the present study, where academic staff expressed concerns about the inconsistent implementation and lack of feedback in performance monitoring systems. This suggests that in Uganda, performance monitoring needs strengthening and standardization to serve as an effective management tool.

Daka (2024) also emphasized the importance of aligning appraisal systems with staff expectations. In his study of private universities in Lusaka, he found mixed staff responses, with some appreciating participatory appraisals and others criticizing the lack of feedback and recognition. The qualitative results of this research echoed similar sentiments, where some deans highlighted irregularity and bias in performance monitoring practices. As recommended by Daka, integrating motivational aspects such as rewards, regular appraisals, and supportive supervision may enhance performance outcomes. Also As Waweru and Kalani (2009) argue in the context of organizational crises, transparent evaluation and accountability mechanisms are essential to prevent systemic inefficiencies—lessons equally applicable to performance monitoring in higher education.

Furthermore, Sabi, Uzoka, and Mlay (2018) brought attention to the technological infrastructure supporting performance systems. They argued that cloud-based technologies can facilitate better collaboration and performance monitoring. While not a direct focus of this research, some deans in the qualitative findings of this study noted the role of technology in tracking performance and providing feedback. However, they also pointed out infrastructural limitations, echoing Sabi et al.’s observations about the challenges faced in developing countries.

Rwothumio et al. (2021) also support these findings. Their study on performance appraisals in Ugandan public universities found a moderately positive correlation between performance appraisals and teaching output (r = 0.452, p < 0.01) and research output (r = 0.379, p < 0.01). This supports the quantitative findings from the present study, which show that academic staff perceived performance monitoring as instrumental in identifying performance gaps and improving alignment with institutional goals.

However, the concerns raised in the qualitative findings regarding inconsistency and lack of feedback are echoed by Atwebembeire et al. (2018), who observed that despite the existence of performance-related policies in Ugandan private universities, limited staff participation and weak follow-up mechanisms undermined performance monitoring efforts. Their study concluded that improved staff involvement in planning and decision-making enhances the quality of teaching and research, indicating a need for a more participatory and consistent monitoring process—an issue also raised by several deans in the present study.

Further support comes from Mohammadi and Karupiah (2020), who found that the quality of work life particularly feedback and clarity of performance expectations significantly influenced academic staff performance. In line with this, the Ugandan academic staff’s call for clearer, consistent, and timely performance feedback reflects the same principle. Performance monitoring is perceived positively when it translates into tangible support and recognition.

Conversely, Yousefi et al. (2020) highlighted that performance monitoring, when poorly implemented, can lead to organizational stress. In their study of Malaysian private universities, stressors like workload and role ambiguity were linked to poor performance. While the Ugandan academic staff did not explicitly report stress, their qualitative responses suggest frustration stemming from unclear processes and the administrative burden associated with monitoring—concerns that may eventually affect performance if unaddressed.

Lastly, Turk and Killumets (2014) noted differing perceptions of performance appraisal systems in Estonian universities. Their findings emphasized the need for flexible and transparent systems that balance institutional goals with staff expectations. This supports the qualitative observation in the current study that rigid and inconsistent monitoring systems may not fully capture staff contributions or support professional development.

The quantitative findings indicated that academic staff in private chartered universities in Western Uganda generally held a favorable perception of performance monitoring. For example, a substantial proportion of respondents agreed that performance monitoring improved accountability (mean = 3.77, SD = 1.02), helped identify performance gaps (mean = 3.65, SD = 0.96), and aligned staff efforts with institutional goals (mean = 3.69, SD = 1.01). However, moderate dissatisfaction was evident regarding the consistency of monitoring processes and the timeliness of feedback (mean = 3.02, SD = 1.11), suggesting room for improvement in communication and implementation practices.

The qualitative results reinforced these findings. Deans reported that while mechanisms for performance monitoring exist, they are often inconsistently applied and lack actionable follow-up. As one respondent highlighted, “We do monitor performance, but the process is sometimes reactive instead of proactive” (Dean 3, DU PT1-31). Another dean added, “There is a lot of paperwork, but follow-up is minimal. The staff would appreciate meaningful feedback” (Dean 7, DU PT1-31). These comments underline the need for a more systematic and continuous approach to performance monitoring.

These results are supported by Molefe (2012), who found that academic staff across institutions in the USA, UK, Australia, Nigeria, and South Africa positively perceived performance monitoring, particularly when aligned with teaching quality and student engagement. Molefe emphasized that appraisal systems focusing on meaningful criteria such as student-teacher interaction and subject clarity are more likely to be accepted and internalized by staff. This reflects the Ugandan context, where staff showed strong agreement with the value of performance monitoring when tied to goal alignment and accountability.

Similarly, Rwothumio et al. (2021), studying Ugandan public universities, revealed a moderately positive correlation between performance appraisal and teaching output (r = 0.452, p < 0.01), as well as research output (r = 0.379, p < 0.01). These correlations underscore the significant role of performance monitoring in enhancing academic functions. This statistical evidence corresponds with the present findings that academic staff believe performance monitoring helps to identify performance gaps and improve results, both in teaching and research.

Nonetheless, the concerns about inconsistency and insufficient feedback mirror findings from Atwebembeire et al. (2018), who reported that many private universities in Uganda lacked robust mechanisms for involving staff in appraisal and feedback processes. Their study showed that only 38% of academic staff felt their performance reviews were followed by constructive dialogue, indicating a gap between policy and practice. This aligns with the present qualitative findings where deans lamented the lack of feedback follow-up and staff involvement in goal-setting and reviews.

The perception that feedback and clarity are critical for improving performance is echoed by Mohammadi and Karupiah (2020), who found a statistically significant relationship (p < 0.05) between work environment factors such as feedback, supervision, and clarity of job expectations with academic staff performance in Malaysian private universities. Their findings affirm the importance of timely, structured performance monitoring systems, as expressed by Ugandan academic staff.

In contrast, Yousefi et al. (2020) revealed that if poorly implemented, performance monitoring could become a source of stress and dissatisfaction. Their study in Malaysia reported that over 60% of staff felt that unclear expectations and ambiguous feedback contributed to job strain. Although the present study’s respondents did not explicitly mention stress, the qualitative narratives about inconsistent monitoring and over-reliance on documentation suggest potential for discontent if not addressed. Employee perceptions of appraisal systems, as shown by Osei and Ackah (2015), can either enhance or undermine motivation, depending on the level of fairness and transparency.

Furthermore, Turk and Killumets (2014) found that rigid and top-down appraisal systems in Estonian universities were often viewed negatively by staff. Their study highlighted the need for more participatory and transparent systems. This insight resonates with the feedback from Ugandan deans advocating for greater inclusion of academic staff in setting performance expectations and developing monitoring frameworks.

In summary, both the quantitative and qualitative findings from this study point to a generally positive perception of performance monitoring among academic staff in private chartered universities in Western Uganda. However, key challenges such as inconsistent implementation, lack of timely feedback, and inadequate staff involvement remain. These findings are corroborated by regional and international literature, which emphasizes the importance of structured, participatory, and feedback-oriented monitoring systems. Addressing these gaps will be essential for enhancing academic productivity, morale, and institutional effectiveness.

Would you like this section formatted for a research article or dissertation chapter?

The quantitative findings revealed that academic staff in private chartered universities in Western Uganda generally held a positive perception of performance monitoring. Specifically, most respondents agreed that performance monitoring improves accountability and aligns academic work with university goals (mean = 3.77, SD = 1.02; mean = 3.69, SD = 1.01, respectively). Respondents also agreed that it helps identify performance gaps (mean = 3.65, SD = 0.96). However, moderate dissatisfaction was observed regarding the frequency and consistency of performance reviews and the availability of timely and actionable feedback (mean = 3.02, SD = 1.11). These results suggest a dual perception: academic staff acknowledge the utility of performance monitoring but also desire improvements in how it is implemented.

Qualitative evidence from interviews with deans reinforced these results. One dean noted, “Performance monitoring exists, but it’s more on paper. Real follow-up is missing, and staff do not get timely feedback” (Dean 2, DU PT1-31). Another dean echoed the same concern: “There is monitoring, but the results are rarely discussed with staff in a constructive way. So, it ends up being a formality” (Dean 5, DU PT1-31). These sentiments suggest that academic staff feel performance monitoring mechanisms lack depth, engagement, and dialogic follow-up. While the structures may be present, their implementation is often superficial or inconsistent.

These findings align with Molefe (2012), who emphasized that academic staff in multiple countries, including the UK, South Africa, and Nigeria, perceived performance monitoring positively when it was based on transparent and fair criteria. Molefe concluded that systems perceived as evaluative but not punitive helped improve staff motivation and performance. This mirrors the Ugandan context, where staff appreciate monitoring efforts when they are geared towards improvement rather than blame.

In addition, Rwothumio et al. (2021) found that in Uganda’s public universities, a moderate positive correlation existed between performance appraisal and academic productivity. Their study recorded a correlation coefficient of r = 0.452 (p < 0.01) for teaching and r = 0.379 (p < 0.01) for research output. These findings reinforce the current study’s quantitative results, where respondents believed that monitoring helps identify performance gaps and enhances professional growth, particularly in research and teaching domains.

However, gaps identified in the current study around timeliness and feedback consistency are supported by Atwebembeire et al. (2018), who found that only 38% of private university academic staff in Uganda believed performance reviews were followed by actionable feedback. The current study’s qualitative findings similarly highlighted that although performance monitoring mechanisms were in place, their actual use for developmental feedback and coaching was limited. As one dean remarked, “There are appraisals, but no one really sits with staff to reflect on how to improve. It’s like ticking boxes” (Dean 6, DU PT1-31).

Moreover, the perception that staff should be involved in setting their performance targets and evaluation indicators emerged in the qualitative findings. One respondent stated, “We are assessed, but we don’t set our goals. Sometimes we are judged on what we were not told to prioritize” (Dean 9, DU PT1-31). This need for participatory appraisal design is echoed by Turk and Killumets (2014), whose study on Estonian universities indicated that top-down performance evaluation frameworks led to dissatisfaction, while participatory systems enhanced buy-in and effectiveness.

Furthermore, the present findings suggest that performance monitoring is often linked to administrative control rather than professional development. Some academic staff felt that monitoring was used more for surveillance than support. For instance, one dean noted, “Sometimes the focus is on what went wrong rather than on how to improve. That demotivates staff” (Dean 7, DU PT1-31). This aligns with Yousefi et al. (2020), who found that performance systems in Malaysian private universities often lacked a developmental orientation, contributing to job stress and disengagement. Over 60% of staff in their study viewed monitoring practices as judgmental rather than growth-oriented.

On the contrary, Mohammadi and Karupiah (2020) found that when performance monitoring was structured around clear expectations, supportive supervision, and timely feedback, it significantly enhanced academic staff performance (p < 0.05). Their findings highlight that monitoring, if well implemented, fosters positive outcomes, which supports the favorable perception of academic staff in the current study regarding accountability and goal alignment.

Another critical issue raised in the qualitative responses relates to the use of ICT in performance monitoring. A few deans mentioned that digital monitoring systems were either underutilized or non-existent: “We rely heavily on manual tracking. An online performance system would make feedback easier and more regular” (Dean 4, DU PT1-31). This finding resonates with Nganyi et al. (2014), who advocated for the adoption of digital monitoring tools to enhance timeliness, accuracy, and accessibility of performance data in academic institutions. The absence of such systems contributes to delays and inconsistencies in feedback, which staff in this study found frustrating.

Lastly, concerns were raised about the link between performance monitoring and reward systems. While staff generally supported monitoring for improvement, they expressed skepticism about whether outstanding performance is recognized or rewarded. As one dean stated, “Even if you excel in your duties, there’s no guarantee of recognition or promotion. That weakens the motivation to perform” (Dean 10, DU PT1-31). This disconnect between appraisal outcomes and reward is also reflected in the literature. Atwebembeire et al. (2018) observed that performance-based rewards in private Ugandan universities were inconsistently applied, undermining the credibility of the monitoring process.

In conclusion, academic staff in private chartered universities in Western Uganda hold a generally favorable view of performance monitoring, particularly in its ability to promote accountability and identify areas for growth. However, both quantitative and qualitative findings highlight critical challenges such as irregular implementation, lack of feedback, insufficient staff involvement, and weak links to reward systems. These findings are in agreement with regional and international studies and point to the need for performance monitoring systems that are participatory, development-oriented, and supported by ICT for real-time engagement. Addressing these gaps will enhance not only academic staff performance but also institutional credibility and effectiveness.

## Conclusions

The findings of this study demonstrate that the perception of academic staff toward performance monitoring in private universities in Western Uganda is moderately positive but marked by concerns about irregular implementation, lack of structured feedback, and limited staff involvement in designing monitoring tools. Quantitative results confirmed a statistically significant positive relationship between perception of performance monitoring and academic staff performance, with regression analysis indicating that perception explained 18.7% of the variance in staff performance (R ^2^ = 0.187, β = 0.394, p < 0.001). Qualitative data revealed that staff were more motivated and productive when performance monitoring was perceived as transparent, participatory, and focused on professional growth rather than fault-finding.

The study concludes that favorable perceptions of performance monitoring are essential in enhancing motivation, accountability, and job performance among academic staff. When academic staff view performance systems as fair and supportive, their work engagement and output increase. Therefore, the perception of performance monitoring is not merely an administrative issue but a critical determinant of institutional effectiveness in private universities.

## 6. Recommendations

1. Standardize Performance Monitoring Procedures:
  Universities should develop clear, fair, and consistent guidelines for performance monitoring to reduce perceptions of bias and irregularity.
2. Enhance Feedback Mechanisms:
  Regular and constructive feedback should be embedded within performance monitoring systems to support continuous professional development.
3. Foster Participatory Appraisal Systems:
  Academic staff should be actively involved in the design and review of performance evaluation tools to promote ownership and relevance.
4. Strengthen Capacity for Monitoring and Evaluation:
  Training programs should be provided to academic leaders and supervisors to ensure effective, ethical, and motivating performance assessment.
5. Leverage Technology for Monitoring:
  Institutions should invest in digital platforms that facilitate transparent tracking of academic outputs and timely dissemination of performance reports.

### Ethical approval statement

This study received ethical approval from the **Research Ethics Committee of Kampala International University,** Uganda. The approval was granted on **September 6** ^**th**^ **2024,** with the reference number KIU-2024-292. The ethics committee approved the research protocol, participant recruitment procedures, and data protection measures and from the Uganda National Council for Science and Technology (UNCST) under national approval number **SS3145ES**. uncst.go.ug

### Informed consent statement

Prior to data collection, all participants were provided with detailed information about the purpose of the study, the procedures involved, their rights as participants, and any potential risks or benefits. Written informed consent was obtained from all participants before their involvement in the study. This consent covered both the questionnaire survey and the interview guide, ensuring that participants understood and agreed to participate voluntarily in either or both aspects of data collection.

## Data Availability

Open Science Framework: Establishing the Perception of Academic Staff on Performance Monitoring in Private Chartered Universities in Western Uganda, https://doi.org/10.17605/OSF.IO/TCRZG ( silaji, T. et al., 2025a). This project contains the following underlying data:
•
*Survey responses.xlsx* (Raw data collected from academic staff survey on performance monitoring and organizational structure). Data is available under the terms of the License: CC0 1.0 Universal Open Science Framework: Establishing the Perception of Academic Staff on Performance Monitoring in Private Chartered Universities in Western Uganda, https://doi.org/10.17605/OSF.IO/TCRZG ( silaji, T. et al., 2025b). This project contains the following extended data:
•
*Questionnaire.pdf* (The full questionnaire used to collect survey data from participants). •
*Interview guide.pdf* ( *Interview transcripts.docx* (Qualitative data from Deans)). • All other key documents that supported this research study Extended data is available under the terms of the License: CC0 1.0 Universal

## References

[ref1] Agyemang CB Ofei SB : Employee work engagement and organizational commitment: A comparative study of public and private sector organizations in Ghana. *European Journal of Business and Management.* 2013;5(24):2222–2839.

[ref25] Atwebembeire J Malunda P : Stakeholder’s perception on financing of private education in Uganda. *Ugandan Journal of Management and Public Policy Studies.* 2018;1(1):79–88.

[ref2] Atwebembeire J Tumwesigye G Ahimbisibwe A : Performance appraisal practices and performance of academic staff in private universities in Uganda: A case study of Bishop Stuart University. *Int. J. Manag. Commerce Innov.* 2018;6(2):1111–1121.

[ref3] Barasa JK Kaabwe SM : Fallacies in policy and strategies of skills training for the informal sector: Evidence from Uganda. *Int. J. Educ. Dev.* 2001;21(1):55–67. 10.1016/S0738-0593(00)00012-6

[ref26] Bell GH Bitton A Desai EV : Health facility management and primary health care performance in Uganda. *BMC Health Serv. Res.* 2018;18(1):275. 10.1186/s12913-022-07674-3 35232451 PMC8886189

[ref27] Christine VH Wanyama S Burton B : Stakeholders, accountability and the theory-practice gap in developing nations’ corporate governance systems: Evidence from Uganda. *Corp. Gov.: Int. Rev.* 2013;13(1):18–38. 10.1108/14720701311302396

[ref4] Daka H : Appraisal systems and academic staff perception in Lusaka’s private universities. *Journal of African Higher Education Management.* 2024;12(1):34–50.

[ref28] Goldberg LR : An alternative “description of personality”: The Big-Five factor structure. * J. Pers. Soc. Psychol.* 1990;59(6):1216–1229. 10.1037/0022-3514.59.6.1216 2283588

[ref5] Ismail AI Raza A : The impact of performance management system on employee performance: A case study of university lecturers in Pakistan. *J. Hum. Resour. Manag. Res.* 2019;2019:1–10. 10.5171/2019.835079

[ref6] Jansen B Smith R : Linking performance feedback with academic staff development: A mixed-methods study. *High. Educ. Res. Dev.* 2021;40(1):120–136. 10.1080/07294360.2020.1799942

[ref29] Kansiime G : Performance management of the academic staffs in Ugandan public and private universities (Doctoral dissertation, Rhodes University). *Rhodes University Institutional Repository.* 2023. Reference Source

[ref7] Kansiime M Singh S : Evaluating performance management systems in Ugandan universities: A comparative analysis of public and private institutions. *African Journal of Educational Management.* 2023;15(1):74–89.

[ref30] Karuhanga BN : Evaluating implementation of strategic performance management practices in universities in Uganda. *Meas. Bus. Excell.* 2015;19(2):42–56. 10.1108/MBE-06-2014-0017

[ref31] Kendra C : The Big Five personality traits. *Verywell Mind.* 2016. Reference Source

[ref8] Kim J Lee S : Academic staff perceptions of performance monitoring and its influence on satisfaction and productivity in South Korean universities. *J. High. Educ. Policy Manag.* 2018;40(3):245–262. 10.1080/1360080X.2018.1462435

[ref9] Mohammadi S Karupiah P : The influence of performance appraisal practices on academic staff performance in Malaysian private higher education institutions. *J. Educ. Soc. Res.* 2020;10(1):142–150. 10.36941/jesr-2020-0013

[ref10] Molefe G : Performance measurement model and academic staff: A survey at selected universities in South Africa. *SA J. Hum. Resour. Manag.* 2012;10(2):1–9. 10.4102/sajhrm.v10i2.326

[ref11] Mutabaruka G : Performance appraisal system and employee productivity in Rwanda’s private universities: A case of Kigali Independent University. *East African Journal of Education and Social Sciences.* 2022;3(1):74–81. 10.46606/eajess2022v03i01.0148

[ref12] Nganyi EW Shigogodi JJ Owano AA : Enhancing performance management in public universities in Kenya through effective information communication technology. *International Journal of Academic Research in Business and Social Sciences.* 2014;4(9):266–278. 10.6007/IJARBSS/v4-i9/1176

[ref13] Nguyen TH Pham HL : Performance monitoring and academic engagement in Vietnamese higher education institutions. *Asian Educ. Dev. Stud.* 2020;9(4):553–570. 10.1108/AEDS-06-2020-0148

[ref14] Osei C Ackah D : Employee perception of performance appraisal system: A case study. *Int. J. Economics Commerce Manag.* 2015;3(1):1–11.

[ref15] Rwothumio JM Aguti JN Nkata JL : Performance appraisal and academic staff productivity in Ugandan public universities: A case study of Kyambogo University. *International Journal of Research and Innovation in Social Science (IJRISS).* 2021;5(2):2454–6186. Reference Source

[ref16] Sabi HM Uzoka F-ME Mlay SV : Cloud computing applications in the education sector: A review of adoption in developing countries. *Educ. Inf. Technol.* 2018;23(2):877–903. 10.1007/s10639-017-9633-4

[ref17] Silaji T Bagiwa ZL Muhammad T : Establishing the Perception of Academic Staff on Performance Monitoring in Private Chartered Universities in Western Uganda. 2025a, July 10. 10.17605/OSF.IO/TCRZG PMC12640481 41287836

[ref18] Silaji T Bagiwa ZL Muhammad T : Establishing the Perception of Academic Staff on Performance Monitoring in Private Chartered Universities in Western Uganda. 2025b, July 23. 10.17605/OSF.IO/TCRZG PMC12640481 41287836

[ref32] Ssemwanga SL Muyinda H : Emotional intelligence competence and job performance of academic staff in private universities in Uganda. *Int. J. Soc. Sci. Humanit. Res.* 2021;9(1):146–153.

[ref19] Thompson L Houghton JD : Performance monitoring perceptions and motivation among academic staff: A Self-Determination Theory perspective. *Educ. Manag. Adm. Lead.* 2019;47(2):292–309. 10.1177/1741143217745886

[ref20] Tibarimbasa AK : *Factors affecting the management of private universities in Uganda.* Makerere University;2010. (Doctoral dissertation). Reference Source

[ref21] Turk M Killumets E : Performance management of academic staff in Estonian universities: Problems and perspectives. *High Educ. Pol.* 2014;27:441–457. 10.1057/hep.2014.20

[ref22] Waweru NM Kalani VM : Commercial banking crises in Kenya: Causes and remedies. *Global Journal of Finance and Banking Issues.* 2009;3(3):23–29.

[ref23] Yousefi R Ahmad J Abdul Razak N : Performance appraisal and job satisfaction among academic staff in Malaysian private universities. *International Journal of Academic Research in Business and Social Sciences.* 2020;10(1):290–306. 10.6007/IJARBSS/v10-i1/6863

[ref24] Zhang Y Wang L : Equity-based performance monitoring and academic staff outcomes in Chinese universities. *Int. J. Educ. Manag.* 2022;36(6):1152–1170. 10.1108/IJEM-09-2021-0395

