# Establishing the Perception of Academic Staff on Performance Monitoring in Private Chartered Universities in Western Uganda

**DOI:** 10.12688/f1000research.167786.3

**Published:** 2025-11-26

**Authors:** Turyamureeba silaji, Zulaihatu Lawal Bagiwa, Tukur Muhammad

**Affiliations:** 1 Department of Educational Foundation, Faculty of Education, Kampala International University - Western Campus, Bushenyi, Western Region, Uganda; 2 Department of Science Education, Faculty of Education, Kampala International University - Western Campus, Bushenyi, Western Region, Uganda

**Keywords:** Performance monitoring, Academic staff Performance, Perception, Private Chartered Universities, Uganda

## Abstract

**Background:**

Effective performance monitoring is essential for improving academic productivity, especially in private higher education institutions. This study aimed to establish the perception of academic staff toward performance monitoring and how these perceptions influence academic staff performance in private chartered universities in Western Uganda. Guided by Expectancy Theory and Self-Determination Theory, the study explored how monitoring practices affect motivation and performance outcomes.

**Methods:**

A convergent parallel mixed-methods design was employed. The quantitative strand involved 386 academic staff selected from five private chartered universities using stratified random sampling. Data were collected using structured questionnaires. In the qualitative strand, semi-structured interviews were conducted with 10 purposively selected Deans of Faculties. Quantitative data were analyzed using descriptive statistics, Pearson correlation, and regression analysis, while qualitative data were analyzed thematically.

**Results:**

Findings revealed that 58.2% of academic staff had a moderately positive perception of existing performance monitoring practices. Pearson correlation analysis showed a moderate positive relationship between perception of performance monitoring and academic staff performance (r = .476, p < 0.01). Regression analysis further indicated that perception of performance monitoring significantly predicted academic staff performance (β = 0.394, p = 0.000), explaining 18.7% of the variance (R ^2^ = 0.187). Qualitative data revealed challenges such as irregular feedback, lack of transparency, and limited involvement of academic staff in developing performance indicators.

**Conclusions:**

The study concludes that academic staff performance can be enhanced when performance monitoring systems are perceived as fair, transparent, and participatory. It recommends the standardization of performance evaluation procedures, improved feedback mechanisms, and the active involvement of academic staff in setting performance targets. Strengthening these areas could improve motivation, engagement, and overall academic performance in private universities across the region.

## Introduction

Performance monitoring in higher education institutions serves as a cornerstone for ensuring quality teaching, research, and service delivery. However, the success of such monitoring systems heavily relies on how academic staff perceive their implementation and intent. This study focuses on establishing the perception of academic staff on performance monitoring in private universities in Western Uganda, a region that has witnessed a growing number of private higher education institutions over the past decade.

This study was guided by Self-Determination Theory ( Deci & Ryan, 2000) and Expectancy Theory (Vroom, 1964), which directly inform the research objectives. The first explores how autonomy, competence, and relatedness shape staff perceptions of monitoring systems, while the latter explains how perceived fairness and expected outcomes drive motivation and performance. These theoretical lenses guided both the quantitative and qualitative analysis, particularly in assessing how perceived feedback, transparency, and participation predict academic performance.

### Background

Employee engagement and commitment are critical factors in organizational performance, and previous studies have shown that both are influenced by how employees perceive appraisal systems ( Agyemang & Ofei, 2013). Also Tibarimbasa (2010) noted persistent management challenges in Uganda’s private universities, including limited staff involvement and weak appraisal frameworks, which continue to affect institutional performance.

Kim and Lee (2018) demonstrated that academic staff’s perceptions of performance monitoring significantly influenced their job satisfaction and productivity, reporting a 20% increase in satisfaction and a 15% boost in productivity. Thompson and Houghton (2019), using Self-Determination Theory, similarly found that staff with favorable perceptions showed a 25% rise in motivation and a 20% increase in performance.

Across East Africa, universities increasingly emphasize performance-based accountability. In Kenya and Tanzania, reforms in the 2010s linked funding to measurable outputs, while Rwanda’s higher education policy ( Mutabaruka, 2022) prioritizes transparent staff evaluation. Uganda’s private universities, however, face fragmented implementation due to limited institutional capacity and weak regulatory oversight ( Kansiime & Singh, 2023). These regional contrasts highlight the importance of contextually adaptive monitoring mechanisms to enhance academic performance.

### Theoretical Framework – Self-Determination Theory (Expanded)

Self-Determination Theory (SDT), developed by Deci and Ryan (1985, 2000), emphasizes that human motivation is influenced by the extent to which three basic psychological needs are satisfied: autonomy, competence, and relatedness.

Autonomy refers to the sense of choice and control over one’s actions.

Competence relates to the perception of being effective and capable in one’s work.

Relatedness reflects the need to feel connected and supported by colleagues and the institution. According to SDT, when performance monitoring systems support these needs, staff are more likely to experience intrinsic motivation, which fosters engagement, persistence, and higher performance. Conversely, systems that are overly controlling, punitive, or opaque undermine these needs, leading to disengagement and lower productivity.

In the context of this study, performance monitoring that involves academic staff in setting performance targets supports autonomy, while constructive and timely feedback enhances competence. Similarly, participatory and transparent evaluation systems strengthen relatedness by building trust between staff and management. Thus, SDT provides a powerful lens to explain why staff perceptions of monitoring practices significantly affect motivation and performance outcomes in private universities.

Nguyen and Pham (2020) highlighted a 30% improvement in performance among Vietnamese staff who perceived performance monitoring as fair and developmental. Jansen and Smith (2021) emphasized the role of structured feedback and career development, while Zhang and Wang (2022), based on Equity Theory, confirmed that fairness in monitoring processes boosts satisfaction and performance. In the Ugandan context, Kansiime and Singh (2023) and Daka (2024) pointed out issues of inconsistent implementation and limited feedback. Finally, Sabi et al. (2018) advocated for the role of technological infrastructure in enhancing performance systems.

Kim and Lee (2018) explored the impact of performance monitoring perceptions on academic staff performance in South Korean universities. Their study aimed to understand how academic staff’s views on performance monitoring systems influence their job satisfaction and productivity. The researchers hypothesized that positive perceptions of performance monitoring would enhance staff performance, while negative perceptions would decrease it. Based on the theory of Motivation by Expectancy, the study employed a cross-sectional survey and quantitative approach, analyzing data through regression analysis and descriptive statistics. The findings revealed that staff who viewed performance monitoring positively experienced a 20% increase in job satisfaction and a 15% improvement in productivity. Conversely, negative perceptions were linked to lower performance and job satisfaction. The study recommended that universities design performance monitoring systems perceived as fair and supportive to boost staff performance and satisfaction. This aligns with Bell et al. (2018) who emphasized the importance of positive performance monitoring perceptions.

Additionally, Thompson and Houghton (2019) explored how academic staff’s perceptions of performance monitoring affect their motivation and performance in private universities. They aimed to analyze the connection between staff views on performance monitoring systems and their levels of motivation and performance. Guided by Self-Determination Theory, the study used a descriptive survey design with a quantitative approach, incorporating structural equation modeling and correlation analysis. The results indicated that favorable perceptions of performance monitoring led to a 25% increase in motivation and a 20% improvement in job performance, while negative perceptions were associated with decreased motivation and performance. The authors recommended implementing transparent and constructive performance monitoring systems to enhance staff motivation and performance. These findings are consistent with those of Kim and Lee (2018), reinforcing the positive impact of favorable performance monitoring perceptions.

In a similar vein, Nguyen and Pham (2020) investigated the effects of performance monitoring systems on academic staff performance and job satisfaction in Vietnamese universities. Their study aimed to assess how staff perceptions influence their performance and satisfaction. Utilizing Performance Appraisal Theory, the researchers conducted a quantitative survey and analyzed the data through factor analysis and multiple regression. The study found that positive perceptions of performance monitoring were associated with a 30% improvement in staff performance and a 25% increase in job satisfaction, while negative perceptions led to lower performance and dissatisfaction. The authors recommended creating performance monitoring systems perceived as fair and beneficial to improve staff performance and satisfaction. This study extends the work of Thompson and Houghton (2019) by providing further evidence of the importance of positive performance monitoring perceptions in enhancing staff outcomes.

Additionally, Jansen and Smith (2021) examined how academic staff in European universities perceive performance monitoring and its impact on their performance. The study sought to analyze the connection between staff perceptions and their performance levels, using Expectancy Theory as a framework. The researchers employed a mixed-methods approach, integrating qualitative thematic analysis with quantitative statistical analysis. Their findings showed that staff with positive perceptions of performance monitoring exhibited a 22% increase in performance, while those with negative perceptions demonstrated reduced performance and engagement. The study recommended improving the design of performance monitoring systems to foster positive staff perceptions and enhance performance. These results support and extend the findings of Nguyen and Pham (2020), further highlighting the critical role of perception in influencing staff performance.

Whereas, Thompson and Houghton (2019) explored how academic staff’s perceptions of performance monitoring influence their motivation and job performance at private universities. The study aimed to understand the connection between these perceptions and staff motivation and performance levels, guided by Self-Determination Theory. Using a descriptive survey design and a quantitative approach, the researchers applied structural equation modeling and correlation analysis. The findings revealed that positive perceptions of performance monitoring led to a 25% boost in motivation and a 20% increase in job performance, whereas negative perceptions resulted in reduced motivation and performance. Additionally, staff who viewed performance monitoring as a career advancement opportunity demonstrated greater commitment and productivity. The authors recommended implementing transparent and constructive performance monitoring systems to enhance staff motivation and performance. These findings are consistent with Kim and Lee (2018) and further emphasize the role of perception in driving staff outcomes. Related to the above, the challenge of aligning higher education practices with national skill needs has long been recognized in Uganda, where policy gaps and strategy inconsistencies have limited the impact of educational reforms ( Barasa & Kaabwe, 2001).

In a similar vein, Nguyen and Pham (2020) examined how performance monitoring systems affect academic staff performance and job satisfaction in Vietnamese universities. Their study aimed to explore the impact of staff perceptions on their performance and satisfaction. Guided by Performance Appraisal Theory, the researchers conducted a quantitative survey and analyzed data using factor analysis and multiple regression. The study found that positive perceptions of performance monitoring were associated with a 30% improvement in staff performance and a 25% increase in job satisfaction, while negative perceptions led to lower performance and dissatisfaction. Additionally, the study highlighted that staff who felt involved in setting performance criteria exhibited greater engagement and better outcomes. The authors recommended creating performance monitoring systems perceived as fair and beneficial to improve staff performance and satisfaction. This study extends the work of Thompson and Houghton (2019) by providing further evidence of the importance of positive performance monitoring perceptions and their impact on engagement and self-improvement.

Adding further depth, Jansen and Smith (2021) investigated academic staff perceptions of performance monitoring and its impact on performance in European universities. The study sought to examine how staff perceptions relate to performance levels, following Expectancy Theory. Researchers employed a mixed-methods approach, integrating qualitative thematic analysis with quantitative statistical techniques. Their findings showed that staff with positive perceptions of performance monitoring exhibited a 22% increase in performance, while those with negative perceptions demonstrated reduced performance and engagement. Notably, institutions that incorporated regular feedback and career development opportunities into their monitoring systems saw enhanced staff performance and satisfaction. The study also noted that performance monitoring systems that included peer evaluations and self-assessments led to a 15% increase in staff morale. The study recommended improving the design of performance monitoring systems to foster positive staff perceptions and enhance performance. These results support and extend the findings of Nguyen and Pham (2020) emphasized the crucial impact of staff perceptions on performance and the additional benefits provided by feedback and development opportunities.

Additionally, Zhang and Wang (2022) investigated the effects of performance monitoring perceptions on academic staff in Chinese universities. Their study aimed to analyze how different types of performance monitoring systems affect staff attitudes and job performance. Guided by the Equity Theory, the researchers conducted a longitudinal study with mixed methods, including surveys and in-depth interviews. The study found that staff who perceived performance monitoring as equitable and supportive showed a 28% increase in job satisfaction and a 22% improvement in job performance. In contrast, those who perceived monitoring as punitive experienced a decrease in job satisfaction and performance. The authors recommended developing performance monitoring systems that emphasize fairness and provide support for professional development. These findings contribute to a broader understanding of how performance monitoring perceptions influence staff outcomes and align with previous research by Jansen and Smith (2021), reinforcing the importance of equity and support in performance monitoring systems.

Related to the above, Kansiime and Singh (2023) noted that many universities worldwide use performance management systems (PMS) as a strategy to improve academic staff performance and enhance teaching and research outputs. However, Ugandan universities have generally neglected the importance of PMS for managing academic staff performance ( Karuhanga, 2015; Atwebembeire and Malunda, 2018). Their study investigated academic staff perceptions of PMS in teaching and research across public and private universities in Uganda. Grounded in performance management theories, the research used a quantitative approach with a structured questionnaire for data collection. Academics from four public and three private universities, selected through non-probability convenience sampling, participated in the study. The results revealed that the average perception of PMS among academic staff was 53.53%, indicating a moderate attitude. Positive attitudes towards PMS were statistically significant in public universities (p = 0.034), while in private universities, the results were statistically insignificant (p = 0.244), reflecting a lack of support for PMS among staff. The study highlights the importance of PMS in universities and suggests the need for further research on its implementation.

Whereas, According to Daka (2024), universities globally are increasingly prioritizing their reputation and quality amidst competitive and dynamic environments. They are tasked with imparting knowledge through skilled academic staff. To achieve this, universities often use performance appraisals to manage staff performance effectively. These appraisals help identify training needs and enhance motivation through feedback. This study explored the perceptions of academic staff on performance appraisals in selected private universities in Lusaka. Using a qualitative approach, data were collected from 30 respondents, including academic staff and supervisors, via open-ended questionnaires and analyzed thematically. The results showed that while participatory appraisals were common, staff perceptions varied. Some staff found appraisals beneficial and noted improvements in performance, whereas others were dissatisfied due to infrequent feedback and a lack of rewards. The study also found that academic staff are more likely to view appraisals positively when they lead to favorable outcomes, though the qualifications of supervisors did not significantly affect staff perceptions. The study recommends aligning appraisal processes with motivational factors to boost staff motivation, performance, and job satisfaction. Similarly, Sabi, Uzoka, and Mlay (2018) emphasize that the advent of cloud computing has led organizations to shift from traditional data center hosting to cloud-based solutions. In Western developed countries, university staff and students have leveraged cloud computing to enhance teaching, research, and collaboration without needing to be physically present on campus. However, universities in developing countries often lack adequate ICT infrastructure. Thus, exploring cloud computing an economical and flexible data storage and transfer solution—could benefit staff in these regions. This study examines how university staff in a developing country perceive the adoption of cloud computing to improve access to ICT resources for educational purposes. Utilizing diffusion of innovation theory and other relevant contextual variables, the study analyzed data from 251 respondents across 11 universities in Uganda using structural equation modeling. The findings highlighted the significant role of socio-cultural factors and perceived results in influencing staff intentions to adopt cloud computing for teaching, research, and collaboration. Additionally, the study revealed notable differences in cloud computing perceptions between male and female staff. The study concludes with recommendations for further research and practical implications.

Furthermore, Molefe (2012) conducted a study to investigate academic staff perceptions of performance measurement across universities in the USA, UK, Australia, Nigeria, and South Africa. The main goal was to create a conceptual model for South African universities that could guide the development of educational policies based on empirical evidence. The study evaluated whether lecturers’ performance could be measured using seven specific dimensions identified in the literature. Molefe highlights the significance of performance management in improving lecturers’ effectiveness. After a thorough literature review, the study concluded that a mixed or integrated performance measurement model could effectively assess both competencies and performance outputs. Statistical analysis of lecturers’ views from the selected universities supported the use of these seven dimensions—knowledge, student-teacher relations, organizational skills, communication skills, subject relevance, assessment procedures, and the utility of assignments—as valid measures of performance, with a Cronbach’s alpha reliability coefficient above 0.70.

Whereas, Rwothumio, Okaka, Kambaza, and Kyomukama (2021) conducted a study on the effectiveness of annual performance appraisals in enhancing lecturers’ performance at public universities in Uganda. Despite these appraisal efforts, issues such as ineffective teaching and low research and publication rates persist, affecting the universities’ ability to contribute to national development. The study explored the relationship between performance appraisals and both teaching and research outputs among academic staff at selected public universities. Employing a mixed-method design with a convergent parallel approach, the researchers collected data from 4 Vice-Chancellors, 4 Directors of Human Resources, and 1,127 full-time academic staff across four purposively selected universities established before 2011. Using stratified random sampling, they surveyed 299 participants, including 291 academic staff, 4 Directors of Human Resources, and 4 Vice-Chancellors. Data collection involved semi-structured questionnaires for academic staff and interview guides for Vice-Chancellors and Directors of Human Resources. Quantitative data were analyzed with Pearson’s correlation, linear regression, and factor analysis, while qualitative data were assessed through thematic content analysis. The results revealed a moderate positive correlation between performance appraisals and teaching output (r = 0.452, p < 0.01), and a moderately positive correlation with research output (r = 0.379, p < 0.01). The study concluded with recommendations for Ugandan public universities to revise their performance appraisal systems to better support the teaching and research roles of academic staff.

While, Kansiime (2023) explored the roles of academic staff across universities globally, emphasizing teaching, research, and administrative duties. Although these core activities are crucial for university success, academic staff in Ugandan universities demonstrate limited commitment and engagement in both teaching and research. The study aimed to assess academic staff performance in Ugandan public and private universities. Drawing on performance theories, the research employed a primarily quantitative methodology and used a structured questionnaire for data collection. Participants were chosen from four public and three private universities out of the 46 in Uganda, using non-probability convenience sampling based on accessibility and availability. The findings revealed a mean teaching skills score of 84.81% and a perceived teaching ability score of 86.34%. However, research skills were rated much lower, averaging 48.30%. These results suggest that academic staff were more involved in teaching than in research activities.

Whereas, Atwebembeire, Namubiru, and Musaazi (2018) investigated the link between staff involvement and the quality of teaching and research in private universities in Uganda. The study was prompted by ongoing concerns about the low quality of graduates and subpar research output from these institutions. Adopting a positivist research approach and a descriptive cross-sectional survey design, data were gathered from four private chartered universities. The sample included 181 lecturers, 23 heads of department, 5 deans, 3 quality assurance officers, 3 research directors, 3 senior staff members from the National Council for Higher Education (NCHE), and 39 student leaders. The data analysis employed descriptive statistics, correlation analysis, and content analysis. The results indicated a significant positive correlation between staff participation and the quality of teaching (r = 0.457, p = 0.000 < 0.05), as well as between staff participation and the quality of research (r = 0.562, p = 0.000 < 0.05). The study concluded that increased staff involvement in planning, implementing activities, and decision-making is associated with improved teaching and research quality. Consequently, the researchers recommended that private universities and the NCHE develop comprehensive policies to boost staff participation in planning and decision-making processes to enhance teaching and research quality.

In a related study, Christine et al. (2013) explored the connection between conflict management and employee performance in selected private universities within Kampala, Uganda. The study aimed to achieve four objectives: to identify respondents’ demographic characteristics such as age, gender, education level, teaching experience, and schedules; to evaluate the extent of conflict management practices in these universities; to assess employee performance; and to determine the relationship between conflict management and employee performance. Data analysis showed that 53.7% of respondents were aged 21-30, while only 1.5% were aged 61-70. The majority were female (58.2%), with males accounting for 41.8%. A notable portion of respondents held a master’s degree (64.7%, or 130 individuals). Full-time respondents numbered 153 (76.1%), compared to 48 (23.9%) part-time respondents. Teaching experience varied, with 134 (66.7%) having 0-5 years, 55 (27.4%) having 6-10 years, and 12 (6.0%) having over 10 years. The study found a positive and significant relationship between conflict management and employee performance, with an r-value of 0.255. The researcher recommended improving academic staff cooperation, offering training on conflict causes, effects, and resolution strategies, creating a supportive work environment, regularly assessing university performance, and motivating staff to enhance their performance.

Based on the above, Mohammadi and Karupiah (2020) investigated how the quality of work life (QWL) affects academic staff performance in universities. They collected data from 379 academic staff members at both public and private universities in Malaysia using a questionnaire. By applying t-tests and one-way ANOVA, they examined variations in QWL and work performance across different demographic factors. Partial Least Squares analysis was used to explore the relationships between various QWL dimensions and performance. The study found that in public universities, factors like feelings of powerlessness and workplace tolerance impacted performance, while in private universities, financial considerations, relationships with co-workers, and workplace tolerance positively affected performance. The results suggest that university managers should concentrate on these key dimensions and enhance them to boost academic staff performance. Similar findings were reported by Ismail and Raza (2019) in Pakistan, where effective performance management systems led to measurable improvements in lecturer performance and motivation.

In line with the above, Yousefi, Devi, and Shuib (2020) observed that private universities in Malaysia have undergone considerable restructuring and program development recently, resulting in increased organizational stress among academic staff. This study aimed to identify and assess the effects of organizational stress indicators on academic performance. Data were gathered using various cluster sampling methods from academic staff at 32 Malaysian private universities, and 190 completed questionnaires were analyzed with SmartPLS software, which provided insights through measurement and structural models. The results showed that workload was the primary stress indicator negatively impacting academic staff performance. Ambiguity and role conflict were identified as secondary and tertiary stressors, respectively, also affecting performance detrimentally. The study contributes significantly to the theoretical and practical understanding of organizational stress in academia and its impact on academic performance. It also offers valuable recommendations for administrators and policymakers in private universities on how to address stress and improve academic staff performance. Similarly, Ssemwanga and Muyinda (2021) conducted research to explore the complexities and misunderstandings related to technical personality traits among part-time academics at private universities. The study specifically investigated how technical personality traits impact job performance for these academics. Technical personality was evaluated based on work competencies linked to two traits from the Big Five Personality Theory: conscientiousness and openness ( Goldberg, 1990; Kendra, 2016). Key competencies examined included intellectual competence, teaching effectiveness, and research supervision skills. Job performance was assessed in terms of task performance, contextual performance, and adaptive performance. Employing a descriptively correlational research design with both quantitative and qualitative methods, the study found that part-time university academics had generally high technical personality competencies, with an average score of 3.43 (SD = 1.15), and their job performance was also high, with an average score of 1.19 (SD = 1.19). These technical competencies explained about 80.3% of the variance in job performance. The study concluded that part-time employees with higher technical competencies tend to demonstrate better job performance.

Furthermore, Turk and Killumets (2014) contend that universities need to optimize their limited resources to effectively prepare a skilled workforce that supports Estonia’s economic strategies. Their study examined academic staff’s expectations and attitudes towards performance appraisals and reward systems. Using surveys and focus-group interviews, the research revealed that although Tartu University employed a performance-based system linking appraisal results to salary, and Tallinn University of Technology used a position-based system where initial salary conditions were key, staff expectations were largely similar across both institutions. University leaders preferred performance-based systems with clear metrics, while most staff members favored more flexible and stable approaches. The study explores the implications of these findings for designing appraisal and reward systems for academic staff.

The structure and performance of universities in Sub-Saharan Africa are deeply influenced by the historical evolution of higher education systems, governance models, and economic realities. In Uganda, the higher education sector has expanded rapidly since liberalisation in the 1990s, leading to the emergence of several private chartered universities. These institutions have become critical actors in expanding access to tertiary education and promoting socio-economic development. However, their governance and performance management practices often mirror mechanistic structures inherited from public systems, characterized by bureaucratic hierarchies, centralised authority, and rigid formalisation. Such structures, while promoting accountability, tend to restrict innovation, collaboration, and academic autonomy ( Adyanga et al., 2022; Abdullahi et al., 2023).

Comparative studies across the East African region reveal similar challenges. In Kenya, for instance, private universities exhibit hierarchical and centralised systems that prioritise compliance with national accreditation standards but often neglect participatory management and academic empowerment. This has led to tensions between administrative efficiency and academic creativity ( Adekoya & Ajagbe, 2021; Adhikari & Shrestha, 2023). In Tanzania and Zambia, the push for performance-based evaluation and accountability has been hindered by limited technological infrastructure and inconsistent performance monitoring mechanisms ( Kabwe, 2024). Similarly, in Nigeria, Abdullahi et al. (2023) found that performance monitoring systems remain largely formalistic, focusing on reporting rather than professional development or feedback-based growth.

In Uganda, private chartered universities face the dual challenge of maintaining quality assurance while fostering staff motivation and productivity. Ahmed and Gohar (2019) observed that most institutions still rely on manual and periodic evaluation systems, which limit the timeliness and transparency of feedback. Consequently, performance monitoring tends to be viewed as a compliance requirement rather than a developmental tool. This situation is compounded by limited funding, inadequate technological infrastructure, and insufficient staff training in performance management systems.

Moreover, the East African Community’s higher education frameworks emphasize harmonisation of academic quality and staff performance standards across member states. Despite these regional efforts, disparities in institutional governance persist. For instance, while some universities have adopted technology-driven monitoring systems, others continue to rely on paper-based reporting and hierarchical oversight ( Adyanga et al., 2022; Kabwe, 2024). The persistence of these disparities underscores the need for more context-specific models of organisational structure and performance monitoring—models that align institutional accountability with staff motivation, innovation, and professional growth.

Therefore, understanding the interaction between organisational structure, performance monitoring, and academic staff performance within private chartered universities in Uganda requires situating the discussion within both national and regional contexts. The present study contributes to this discourse by examining how structural rigidity and limited feedback mechanisms influence academic productivity, while proposing evidence-based strategies to enhance performance management systems that are both developmental and contextually relevant to East Africa’s higher education landscape.

## Methods

A convergent parallel mixed-methods design was employed to collect and analyze both quantitative and qualitative data simultaneously. The study involved:

Participants: 386 academic staff surveyed using structured questionnaires and 10 deans of faculties interviewed through semi-structured guides. Academic staff were selected from two private chartered universities using a stratified random sampling procedure. First, the population was stratified by faculty to ensure proportional representation across disciplines. Within each stratum, staff were randomly selected using staff lists provided by university registrars. A total of 450 questionnaires were distributed, of which 386 were returned fully completed, yielding a response rate of 85.8%. This high rate enhances the representativeness and reliability of the quantitative findings. For the qualitative strand, 10 Deans of Faculties were purposively selected based on their leadership role and experience in performance monitoring practices.

Ten Deans of Faculties were purposively selected based on their central administrative roles in overseeing teaching, research, and performance monitoring. This group was ideal because deans serve as institutional mediators between university management and academic staff, offering strategic insight into how performance monitoring policies are implemented in practice. The sample size aligns with Guest, Namey, and Chen (2020), who suggest that thematic saturation in homogeneous qualitative samples is typically achieved with 6–12 interviews.

Sampling: Academic staff were selected from five private chartered universities using stratified random sampling.

### Instrument validity and reliability

*Quantitative strand (questionnaire):*

The questionnaire was developed based on prior instruments used in higher education performance monitoring research (e.g., Kim & Lee, 2018; Nguyen & Pham, 2020), and was adapted to the Ugandan private university context. To ensure content validity, three experts in higher education management and organizational psychology reviewed the items for clarity, relevance, and coverage of the construct domains (teaching, research, service, and appraisal processes). Adjustments were made following their feedback to refine item wording and eliminate redundancy. Construct validity was examined through exploratory factor analysis (EFA), which confirmed that items loaded strongly on their intended dimensions, with factor loadings exceeding 0.50. To test reliability, internal consistency was assessed using Cronbach’s alpha. The coefficients for the main scales ranged between 0.79 and 0.86, which are above the recommended threshold of 0.70, indicating good reliability. A pilot test with 30 academic staff not included in the final sample further demonstrated clarity and usability of the instrument.

*Qualitative strand (interview guide):*

The semi-structured interview guide was designed to explore deeper perspectives on performance monitoring practices, complementing the survey data. Face validity was ensured by circulating the draft guide among two senior qualitative researchers and two deans of faculties, who confirmed that the questions were clear, contextually appropriate, and aligned with the study’s objectives. Content validity was further established by aligning the interview items with the guiding theoretical framework (Self-Determination Theory and Expectancy Theory) to ensure that key constructs such as autonomy, feedback, fairness, and motivation were addressed. To enhance credibility and dependability, the guide was pilot tested with two academic leaders from institutions outside the study sample. Minor adjustments were made to improve question flow and neutrality. During data collection, reliability was supported through the use of a consistent interview protocol, audio recordings, and verbatim transcription. To strengthen trustworthiness, data were subjected to member checking (participants reviewed summaries of their responses for accuracy) and triangulation with quantitative findings.

Together, these measures ensured that both the quantitative and qualitative instruments were valid, reliable, and fit for purpose in the Ugandan private university context.

### Data collection

Quantitative: Structured questionnaires measured staff perceptions across several dimensions of performance monitoring.

Qualitative: Interviews captured in-depth perspectives on implementation practices and challenges.

Data Analysis:

Quantitative: Data analyzed using SPSS for descriptive statistics, Pearson correlation, and regression analysis.

Qualitative: Thematic analysis identified recurring themes related to transparency, feedback, and staff involvement.

## Results

Quantitative findings revealed that only 42% of respondents agreed that performance monitoring was regularly conducted, and just 38% felt it was fair and transparent. A further 61% indicated dissatisfaction with feedback mechanisms. Qualitative data supported these findings: several deans reported irregular appraisal processes, lack of standardized monitoring tools, and insufficient institutional support.

### Interpretation of Results of Table 1

**Table 1.** Showing perception of academic staff on performance monitoring.

S/N	Perception of academic staff on performance monitoring	F (%)	SD	D	N	A	SA	Mean	STD
B3.1	The performance monitoring system accurately evaluates my teaching performance.	F (%)	8 (2.1)	17 (4.4)	49 (12.7)	208 (53.9)	104 (26.9)	3.99	0.87
B3.2	The performance monitoring system accurately evaluates my research contributions.	F (%)	13 (3.4)	31 (8.0)	49 (12.7)	182 (47.2)	111 (28.8)	3.90	1.01
B3.3	The performance monitoring system accurately evaluates my service and administrative roles.	F (%)	14 (3.6)	23 (6.0)	50 (13.0)	179 (46.4)	120 (31.1)	3.95	1.00
B3.4	The performance criteria are relevant to my academic responsibilities.	F (%)	8 (2.1)	22 (5.7)	66 (17.1)	188 (48.7)	102 (26.4)	3.91	0.92
B3.5	Performance monitoring helps identify my strengths and areas for improvement.	F (%)	13 (3.4)	33 (8.5)	54 (14.0)	178 (46.1)	108 (28.0)	3.87	1.02
	**Grand mean**							**3.92**	

The result of whether the performance monitoring system accurately evaluates teaching performance was that the majority agreed (53.9%) or strongly agreed (26.9%) that the system does so, while (2.1%) strongly disagree or (4.4%) disagree and (12.7%) remained neutral. A mean of 3.99 indicates general agreement and an STD of 0.87 shows moderate response consistency. To confirm if a performance monitoring system can accurately evaluate research contribution, the results are similar to those of B3.1, where a significant portion agreed (47.2%) or strongly agreed (28.8%), and (3.4%) strongly disagree or (8.0%) disagree though (12.7%) were neutral. Mean: 3.90, indicating positive perceptions, and STD: 1.01, reflecting more variability compared to B3.1.

Accurate evaluation of service and administrative roles results shows that a notable 46.4% agreed, and 31.1% strongly agreed, with low disagreement (3.6%) strongly disagree or (6.0%) disagree. Mean: 3.95, showing positive agreement, and STD is 1.00, indicating moderate consistency. The result of the performance criteria relevant to academic responsibilities showed that the majority agreed (48.7%) or strongly agreed (26.4%) and (2.1%) strongly disagree or (5.7%) disagree on the relevance of performance criteria though (17.1%) remained neutral. The mean is 3.91, suggesting favorable perceptions, and the STD of 0.92, implying moderate consistency. Finally, on whether performance monitoring helps identify strengths and areas for improvement of academic staff, a total of (46.1%) agreed (28.0%) strongly agreed, while (8.5%) disagreed, (3.4%) strongly agreed or 8.5% disagree) though (14.0%) remained undecided. Mean: 3.87, indicating general agreement but slightly lower than others. STD is 1.02, the highest variability among all items.

The grand mean across all items is approximately **3.92**, indicating that academic staff generally agree with the statements about the performance monitoring system.

This section presents the findings on the four key constructs of performance monitoring evaluation methods, frequency of monitoring, feedback and reporting, and use of technology and their relationship with academic staff performance in private chartered universities in Western Uganda. Both quantitative and qualitative data were analyzed to provide a comprehensive understanding of staff perceptions and institutional practices.

The first construct, evaluation methods, assessed the accuracy and relevance of the systems used to appraise teaching, research, and administrative performance. Quantitative analysis revealed a strong positive correlation between evaluation methods and academic staff performance (r = 0.63, p < 0.001). Regression analysis indicated that evaluation methods were a significant predictor of performance (β = 0.22, t = 4.50, p < 0.001), suggesting that robust and well-structured evaluation frameworks enhance staff productivity and professional effectiveness. Qualitative interviews reinforced this finding. Many Deans emphasized the importance of systematic evaluation in aligning staff outputs with institutional expectations. One participant noted, “The evaluation system clearly outlines criteria for teaching, research, and administrative duties, which motivates staff to perform better” (DU PT4-272). Another highlighted that “evaluation methods help identify areas of strength and those requiring improvement, providing a roadmap for development” (DU PT5-279). However, some respondents reported inconsistencies, indicating that subjective interpretations occasionally reduce credibility. These findings echo Ahmed and Gohar (2019), who observed that structured performance monitoring enhances accountability and staff motivation in higher education institutions. Similarly, Abdulrahaman (2020) emphasized that transparent and standardized evaluation procedures are critical for improving academic staff performance in Nigerian universities.

The second construct, frequency of monitoring, measured how regularly academic staff performance is assessed. Results showed a strong positive correlation (r = 0.60, p < 0.001), with regression analysis confirming that frequent monitoring significantly predicts staff performance (β = 0.20, t = 3.57, p < 0.001). These results suggest that regular assessments facilitate timely feedback, enabling staff to correct shortcomings and reinforce best practices promptly. Qualitative data further highlighted that consistent monitoring encourages discipline and proactive engagement. As one Dean explained, “Frequent performance reviews ensure that staff are aware of expectations and remain focused on achieving targets” (DU PT2-274). Another participant remarked that “regular monitoring prevents complacency and promotes continuous professional growth” (DU PT6-278). Nonetheless, some respondents cautioned that excessive monitoring can induce stress, especially when evaluation periods coincide with heavy workloads. This finding aligns with Adams and Wang (2020), who reported that while frequent performance appraisals enhance accountability, they may also contribute to burnout if not balanced with support mechanisms.

The third construct, feedback and reporting, focused on the mechanisms used to communicate performance results and guide staff development. Quantitative analysis indicated a moderate positive correlation with academic staff performance (r = 0.58, p < 0.001), and regression analysis showed it was a significant predictor (β = 0.18, t = 3.83, p < 0.001). These findings suggest that structured feedback and clear reporting channels foster accountability and professional improvement.

Qualitative findings revealed that constructive feedback enhances teaching practices, research output, and administrative efficiency. One Dean stated, “Positive, actionable feedback has improved my teaching strategies and strengthened collaboration with colleagues” (DU PT1-313). Another emphasized, “Timely reporting allows us to track our progress and adjust priorities accordingly” (DU PT2-314). However, negative experiences emerged when feedback was inconsistent or perceived as punitive. “Criticism without explanation once caused tension in my department” (DU PT5-317), reported one Dean. This underscores the need for feedback to be objective, transparent, and developmental. These observations are supported by Kabwe (2024), who highlighted that feedback mechanisms are instrumental in promoting trust, engagement, and performance in higher education institutions.

The grand mean across all performance monitoring constructs was 3.92, indicating that academic staff generally agree that evaluation methods, frequency of monitoring, feedback and reporting, and use of technology positively influence their performance. The overall correlation between performance monitoring and academic staff performance was strong (r = 0.66, p < 0.001), with regression results showing that all four constructs collectively explain 41% of the variance in performance (R ^2^ = 0.41, F = 34.20, p < 0.001). These results underscore the importance of a comprehensive, structured, and technology-enabled performance monitoring system in private universities. When implemented consistently, such systems enhance accountability, motivation, professional growth, and institutional effectiveness. However, qualitative findings indicate that challenges related to infrastructure, workload, and feedback consistency must be addressed to maximize the developmental potential of performance monitoring.

In conclusion, the integration of evaluation methods, monitoring frequency, feedback mechanisms, and technology represents a holistic approach that significantly enhances academic staff performance. Aligning these constructs with institutional support, capacity building, and participatory implementation can transform performance monitoring from a bureaucratic exercise into a strategic tool for academic excellence, innovation, and sustained organizational growth, as supported by Ahmed & Gohar (2019); Abdullahi et al. (2023); Adams & Wang (2020); and Alhassan & Habib (2024).

## Discussion

The study’s findings align with Kim and Lee (2018), Nguyen and Pham (2020), and Zhang and Wang (2022) by demonstrating that positive perceptions of monitoring are linked with increased performance and satisfaction. Respondents in this study echoed similar sentiments, stressing the need for fairness, feedback, and involvement in designing monitoring criteria. Conversely, the findings also mirror concerns raised by Kansiime and Singh (2023) and Daka (2024) regarding inconsistencies and negative perceptions in performance monitoring systems. In Rwanda, Mutabaruka (2022) found that clear appraisal processes significantly improved academic staff productivity, underscoring the relevance of well-structured systems across East Africa.

The positive correlation between perceptions of fairness and performance aligns with Expectancy Theory’s principle that motivation increases when outcomes are perceived as attainable and valued. Similarly, qualitative findings showing a need for autonomy and competence reflect Self-Determination Theory’s assertion that intrinsic motivation thrives in supportive environments. 

The findings of the current study align strongly with the conclusions drawn by Kim and Lee (2018), who established that academic staff’s perceptions of performance monitoring significantly influenced their job satisfaction and productivity in South Korean universities. Kim and Lee reported that positive perceptions were associated with a 20% increase in satisfaction and a 15% boost in productivity. Similarly, the present study revealed that academic staff who perceived performance monitoring positively demonstrated higher levels of motivation and performance. This supports the theory of motivation by expectancy, suggesting that favorable perceptions act as catalysts for increased performance. The present findings reinforce the notion that performance monitoring systems must be seen as fair, supportive, and motivating.

The findings strongly align with Expectancy Theory, which posits that staff motivation depends on the perceived fairness and usefulness of performance monitoring systems. Respondents who viewed monitoring as transparent and linked to meaningful outcomes reported higher motivation and productivity, reflecting expectancy-value mechanisms. Similarly, the qualitative themes of autonomy, competence, and relatedness map directly onto Self-Determination Theory. For instance, the lack of involvement in setting performance targets was seen as undermining autonomy, while irregular feedback limited staff’s sense of competence. These insights highlight how theoretical frameworks not only guided the study but also provide explanatory depth for the observed results ( Deci & Ryan, 2000).

In line with these results, Thompson and Houghton (2019) also found a significant link between positive performance monitoring perceptions and improved motivation and job performance among academic staff in private universities. Their study, based on Self-Determination Theory, revealed that staff with favorable perceptions showed a 25% rise in motivation and a 20% increase in performance. The current research similarly observed that when staff viewed performance monitoring as fair and transparent, their motivation and work engagement improved. This reflects the importance of aligning performance monitoring mechanisms with motivational principles to foster desirable outcomes in academic staff.

Nguyen and Pham (2020) further affirmed this correlation, indicating that positive perceptions of performance monitoring in Vietnamese universities led to a 30% improvement in performance and a 25% rise in job satisfaction. The findings of this research are consistent with theirs, showing that academic staff who viewed monitoring systems as fair and developmental were more engaged and productive. Furthermore, Nguyen and Pham highlighted the importance of involving staff in developing performance criteria—a sentiment echoed by respondents in this study, who emphasized the need for participatory and transparent performance evaluation processes.

Jansen and Smith (2021) also underscore this trend, finding that academic staff with positive perceptions of performance monitoring exhibited a 22% increase in performance. Their mixed-methods approach revealed that structured feedback and career development opportunities embedded within monitoring systems enhanced staff outcomes. These insights resonate with this study’s qualitative findings, where deans and academic staff emphasized the importance of feedback, goal-setting, and supportive structures within the performance monitoring process. This reinforces the view that perception is not only a subjective element but also a predictor of professional engagement and output.

Similarly, Zhang and Wang (2022), using Equity Theory, found that equitable and supportive monitoring systems significantly boosted job satisfaction and performance in Chinese universities. Conversely, punitive monitoring reduced these outcomes. The current study supports these findings, as many respondents expressed that performance monitoring perceived as controlling or fault-finding demoralized staff. This highlights the critical need for universities to design systems that prioritize fairness and developmental support over mere evaluation.

In the Ugandan context, Kansiime and Singh (2023) noted that performance management systems were underutilized or improperly implemented, especially in private universities. Their research revealed that while public university staff had moderately positive perceptions of PMS (p = 0.034), private university staff perceptions were statistically insignificant (p = 0.244). These insights align with findings from the present study, where academic staff expressed concerns about the inconsistent implementation and lack of feedback in performance monitoring systems. This suggests that in Uganda, performance monitoring needs strengthening and standardization to serve as an effective management tool.

Daka (2024) also emphasized the importance of aligning appraisal systems with staff expectations. In his study of private universities in Lusaka, he found mixed staff responses, with some appreciating participatory appraisals and others criticizing the lack of feedback and recognition. The qualitative results of this research echoed similar sentiments, where some deans highlighted irregularity and bias in performance monitoring practices. As recommended by Daka, integrating motivational aspects such as rewards, regular appraisals, and supportive supervision may enhance performance outcomes. Also As Waweru and Kalani (2009) argue in the context of organizational crises, transparent evaluation and accountability mechanisms are essential to prevent systemic inefficiencies—lessons equally applicable to performance monitoring in higher education.

Furthermore, Sabi, Uzoka, and Mlay (2018) brought attention to the technological infrastructure supporting performance systems. They argued that cloud-based technologies can facilitate better collaboration and performance monitoring. While not a direct focus of this research, some deans in the qualitative findings of this study noted the role of technology in tracking performance and providing feedback. However, they also pointed out infrastructural limitations, echoing Sabi et al.’s observations about the challenges faced in developing countries.

Rwothumio et al. (2021) also support these findings. Their study on performance appraisals in Ugandan public universities found a moderately positive correlation between performance appraisals and teaching output (r = 0.452, p < 0.01) and research output (r = 0.379, p < 0.01). This supports the quantitative findings from the present study, which show that academic staff perceived performance monitoring as instrumental in identifying performance gaps and improving alignment with institutional goals.

However, the concerns raised in the qualitative findings regarding inconsistency and lack of feedback are echoed by Atwebembeire et al. (2018), who observed that despite the existence of performance-related policies in Ugandan private universities, limited staff participation and weak follow-up mechanisms undermined performance monitoring efforts. Their study concluded that improved staff involvement in planning and decision-making enhances the quality of teaching and research, indicating a need for a more participatory and consistent monitoring process—an issue also raised by several deans in the present study.

Further support comes from Mohammadi and Karupiah (2020), who found that the quality of work life particularly feedback and clarity of performance expectations significantly influenced academic staff performance. In line with this, the Ugandan academic staff’s call for clearer, consistent, and timely performance feedback reflects the same principle. Performance monitoring is perceived positively when it translates into tangible support and recognition.

Conversely, Yousefi et al. (2020) highlighted that performance monitoring, when poorly implemented, can lead to organizational stress. In their study of Malaysian private universities, stressors like workload and role ambiguity were linked to poor performance. While the Ugandan academic staff did not explicitly report stress, their qualitative responses suggest frustration stemming from unclear processes and the administrative burden associated with monitoring—concerns that may eventually affect performance if unaddressed.

Lastly, Turk and Killumets (2014) noted differing perceptions of performance appraisal systems in Estonian universities. Their findings emphasized the need for flexible and transparent systems that balance institutional goals with staff expectations. This supports the qualitative observation in the current study that rigid and inconsistent monitoring systems may not fully capture staff contributions or support professional development.

The quantitative findings indicated that academic staff in private chartered universities in Western Uganda generally held a favorable perception of performance monitoring. For example, a substantial proportion of respondents agreed that performance monitoring improved accountability (mean = 3.77, SD = 1.02), helped identify performance gaps (mean = 3.65, SD = 0.96), and aligned staff efforts with institutional goals (mean = 3.69, SD = 1.01). However, moderate dissatisfaction was evident regarding the consistency of monitoring processes and the timeliness of feedback (mean = 3.02, SD = 1.11), suggesting room for improvement in communication and implementation practices.

The qualitative results reinforced these findings. Deans reported that while mechanisms for performance monitoring exist, they are often inconsistently applied and lack actionable follow-up. As one respondent highlighted, “We do monitor performance, but the process is sometimes reactive instead of proactive” (Dean 3, DU PT1-31). Another dean added, “There is a lot of paperwork, but follow-up is minimal. The staff would appreciate meaningful feedback” (Dean 7, DU PT1-31). These comments underline the need for a more systematic and continuous approach to performance monitoring.

These results are supported by Molefe (2012), who found that academic staff across institutions in the USA, UK, Australia, Nigeria, and South Africa positively perceived performance monitoring, particularly when aligned with teaching quality and student engagement. Molefe emphasized that appraisal systems focusing on meaningful criteria such as student-teacher interaction and subject clarity are more likely to be accepted and internalized by staff. This reflects the Ugandan context, where staff showed strong agreement with the value of performance monitoring when tied to goal alignment and accountability.

Similarly, Rwothumio et al. (2021), studying Ugandan public universities, revealed a moderately positive correlation between performance appraisal and teaching output (r = 0.452, p < 0.01), as well as research output (r = 0.379, p < 0.01). These correlations underscore the significant role of performance monitoring in enhancing academic functions. This statistical evidence corresponds with the present findings that academic staff believe performance monitoring helps to identify performance gaps and improve results, both in teaching and research.

Nonetheless, the concerns about inconsistency and insufficient feedback mirror findings from Atwebembeire et al. (2018), who reported that many private universities in Uganda lacked robust mechanisms for involving staff in appraisal and feedback processes. Their study showed that only 38% of academic staff felt their performance reviews were followed by constructive dialogue, indicating a gap between policy and practice. This aligns with the present qualitative findings where deans lamented the lack of feedback follow-up and staff involvement in goal-setting and reviews.

The perception that feedback and clarity are critical for improving performance is echoed by Mohammadi and Karupiah (2020), who found a statistically significant relationship (p < 0.05) between work environment factors such as feedback, supervision, and clarity of job expectations with academic staff performance in Malaysian private universities. Their findings affirm the importance of timely, structured performance monitoring systems, as expressed by Ugandan academic staff.

In contrast, Yousefi et al. (2020) revealed that if poorly implemented, performance monitoring could become a source of stress and dissatisfaction. Their study in Malaysia reported that over 60% of staff felt that unclear expectations and ambiguous feedback contributed to job strain. Although the present study’s respondents did not explicitly mention stress, the qualitative narratives about inconsistent monitoring and over-reliance on documentation suggest potential for discontent if not addressed. Employee perceptions of appraisal systems, as shown by Osei and Ackah (2015), can either enhance or undermine motivation, depending on the level of fairness and transparency.

Furthermore, Turk and Killumets (2014) found that rigid and top-down appraisal systems in Estonian universities were often viewed negatively by staff. Their study highlighted the need for more participatory and transparent systems. This insight resonates with the feedback from Ugandan deans advocating for greater inclusion of academic staff in setting performance expectations and developing monitoring frameworks.

In summary, both the quantitative and qualitative findings from this study point to a generally positive perception of performance monitoring among academic staff in private chartered universities in Western Uganda. However, key challenges such as inconsistent implementation, lack of timely feedback, and inadequate staff involvement remain. These findings are corroborated by regional and international literature, which emphasizes the importance of structured, participatory, and feedback-oriented monitoring systems. Addressing these gaps will be essential for enhancing academic productivity, morale, and institutional effectiveness.

Would you like this section formatted for a research article or dissertation chapter?

The quantitative findings revealed that academic staff in private chartered universities in Western Uganda generally held a positive perception of performance monitoring. Specifically, most respondents agreed that performance monitoring improves accountability and aligns academic work with university goals (mean = 3.77, SD = 1.02; mean = 3.69, SD = 1.01, respectively). Respondents also agreed that it helps identify performance gaps (mean = 3.65, SD = 0.96). However, moderate dissatisfaction was observed regarding the frequency and consistency of performance reviews and the availability of timely and actionable feedback (mean = 3.02, SD = 1.11). These results suggest a dual perception: academic staff acknowledge the utility of performance monitoring but also desire improvements in how it is implemented.

Qualitative evidence from interviews with deans reinforced these results. One dean noted, “Performance monitoring exists, but it’s more on paper. Real follow-up is missing, and staff do not get timely feedback” (Dean 2, DU PT1-31). Another dean echoed the same concern: “There is monitoring, but the results are rarely discussed with staff in a constructive way. So, it ends up being a formality” (Dean 5, DU PT1-31). These sentiments suggest that academic staff feel performance monitoring mechanisms lack depth, engagement, and dialogic follow-up. While the structures may be present, their implementation is often superficial or inconsistent.

These findings align with Molefe (2012), who emphasized that academic staff in multiple countries, including the UK, South Africa, and Nigeria, perceived performance monitoring positively when it was based on transparent and fair criteria. Molefe concluded that systems perceived as evaluative but not punitive helped improve staff motivation and performance. This mirrors the Ugandan context, where staff appreciate monitoring efforts when they are geared towards improvement rather than blame.

In addition, Rwothumio et al. (2021) found that in Uganda’s public universities, a moderate positive correlation existed between performance appraisal and academic productivity. Their study recorded a correlation coefficient of r = 0.452 (p < 0.01) for teaching and r = 0.379 (p < 0.01) for research output. These findings reinforce the current study’s quantitative results, where respondents believed that monitoring helps identify performance gaps and enhances professional growth, particularly in research and teaching domains.

However, gaps identified in the current study around timeliness and feedback consistency are supported by Atwebembeire et al. (2018), who found that only 38% of private university academic staff in Uganda believed performance reviews were followed by actionable feedback. The current study’s qualitative findings similarly highlighted that although performance monitoring mechanisms were in place, their actual use for developmental feedback and coaching was limited. As one dean remarked, “There are appraisals, but no one really sits with staff to reflect on how to improve. It’s like ticking boxes” (Dean 6, DU PT1-31).

Moreover, the perception that staff should be involved in setting their performance targets and evaluation indicators emerged in the qualitative findings. One respondent stated, “We are assessed, but we don’t set our goals. Sometimes we are judged on what we were not told to prioritize” (Dean 9, DU PT1-31). This need for participatory appraisal design is echoed by Turk and Killumets (2014), whose study on Estonian universities indicated that top-down performance evaluation frameworks led to dissatisfaction, while participatory systems enhanced buy-in and effectiveness.

Furthermore, the present findings suggest that performance monitoring is often linked to administrative control rather than professional development. Some academic staff felt that monitoring was used more for surveillance than support. For instance, one dean noted, “Sometimes the focus is on what went wrong rather than on how to improve. That demotivates staff” (Dean 7, DU PT1-31). This aligns with Yousefi et al. (2020), who found that performance systems in Malaysian private universities often lacked a developmental orientation, contributing to job stress and disengagement. Over 60% of staff in their study viewed monitoring practices as judgmental rather than growth-oriented.

On the contrary, Mohammadi and Karupiah (2020) found that when performance monitoring was structured around clear expectations, supportive supervision, and timely feedback, it significantly enhanced academic staff performance (p < 0.05). Their findings highlight that monitoring, if well implemented, fosters positive outcomes, which supports the favorable perception of academic staff in the current study regarding accountability and goal alignment.

Another critical issue raised in the qualitative responses relates to the use of ICT in performance monitoring. A few deans mentioned that digital monitoring systems were either underutilized or non-existent: “We rely heavily on manual tracking. An online performance system would make feedback easier and more regular” (Dean 4, DU PT1-31). This finding resonates with Nganyi et al. (2014), who advocated for the adoption of digital monitoring tools to enhance timeliness, accuracy, and accessibility of performance data in academic institutions. The absence of such systems contributes to delays and inconsistencies in feedback, which staff in this study found frustrating.

The interviews with Deans provided deeper insights into the challenges of performance monitoring. One dean noted, “Performance monitoring exists, but it is often a formality. Staff rarely receive constructive feedback that could help them improve” (Dean 2). Another emphasized limited staff participation: “We are assessed, but academic staff are not involved in setting the goals against which they are evaluated” (Dean 9). Several respondents described monitoring as reactive rather than proactive: “Follow-up is minimal; monitoring only happens when problems arise” (Dean 3). These verbatim accounts reinforce the quantitative results by demonstrating how lack of feedback, transparency, and participation undermine the perceived effectiveness of monitoring systems.

Lastly, concerns were raised about the link between performance monitoring and reward systems. While staff generally supported monitoring for improvement, they expressed skepticism about whether outstanding performance is recognized or rewarded. As one dean stated, “Even if you excel in your duties, there’s no guarantee of recognition or promotion. That weakens the motivation to perform” (Dean 10, DU PT1-31). This disconnect between appraisal outcomes and reward is also reflected in the literature. Atwebembeire et al. (2018) observed that performance-based rewards in private Ugandan universities were inconsistently applied, undermining the credibility of the monitoring process.

In conclusion, academic staff in private chartered universities in Western Uganda hold a generally favorable view of performance monitoring, particularly in its ability to promote accountability and identify areas for growth. However, both quantitative and qualitative findings highlight critical challenges such as irregular implementation, lack of feedback, insufficient staff involvement, and weak links to reward systems. These findings are in agreement with regional and international studies and point to the need for performance monitoring systems that are participatory, development-oriented, and supported by ICT for real-time engagement. Addressing these gaps will enhance not only academic staff performance but also institutional credibility and effectiveness.

## Conclusions

The findings of this study demonstrate that the perception of academic staff toward performance monitoring in private universities in Western Uganda is moderately positive but marked by concerns about irregular implementation, lack of structured feedback, and limited staff involvement in designing monitoring tools. Quantitative results confirmed a statistically significant positive relationship between perception of performance monitoring and academic staff performance, with regression analysis indicating that perception explained 18.7% of the variance in staff performance (R ^2^ = 0.187, β = 0.394, p < 0.001). Qualitative data revealed that staff were more motivated and productive when performance monitoring was perceived as transparent, participatory, and focused on professional growth rather than fault-finding.

The study concludes that favorable perceptions of performance monitoring are essential in enhancing motivation, accountability, and job performance among academic staff. When academic staff view performance systems as fair and supportive, their work engagement and output increase. Therefore, the perception of performance monitoring is not merely an administrative issue but a critical determinant of institutional effectiveness in private universities.

This study demonstrated that academic staff in private chartered universities in Western Uganda generally hold moderately positive perceptions of performance monitoring, yet significant concerns remain regarding irregular implementation, lack of structured feedback, and limited staff involvement in the design of monitoring tools. Quantitative results confirmed a statistically significant positive relationship between perceptions of monitoring and staff performance, while qualitative insights highlighted systemic weaknesses such as reactive rather than proactive monitoring and limited recognition of staff contributions.

Anchoring the findings in Expectancy Theory, the study shows that when monitoring processes are perceived as fair, transparent, and linked to meaningful outcomes, academic staff are more motivated and productive. Similarly, Self-Determination Theory provides explanatory depth: perceptions of limited autonomy, insufficient feedback undermining competence, and weak collegial support all reduced intrinsic motivation. Conversely, where autonomy, competence, and relatedness were supported, staff performance and engagement improved.

The study makes two key contributions. First, it offers empirical evidence from a Ugandan private university context, filling a gap in higher education literature where performance monitoring is often underexplored. Second, it strengthens theoretical integration by showing how SDT and Expectancy Theory explain the mechanisms through which perceptions of monitoring shape staff performance.

In practice, the results underline the need for performance monitoring systems that are standardized, feedback-driven, participatory, and ethically grounded. If private universities in Uganda design appraisal systems that promote autonomy, competence, and fairness, academic staff will not only perform better but also contribute more meaningfully to teaching, research, and institutional growth.

## 6. Recommendations

1. Standardize Performance Monitoring Procedures:
  Universities should develop clear, fair, and consistent guidelines for performance monitoring to reduce perceptions of bias and irregularity.
2. Enhance Feedback Mechanisms:
  Regular and constructive feedback should be embedded within performance monitoring systems to support continuous professional development.
3. Foster Participatory Appraisal Systems:
  Academic staff should be actively involved in the design and review of performance evaluation tools to promote ownership and relevance.
4. Strengthen Capacity for Monitoring and Evaluation:
  Training programs should be provided to academic leaders and supervisors to ensure effective, ethical, and motivating performance assessment.
5. Leverage Technology for Monitoring:
  Institutions should invest in digital platforms that facilitate transparent tracking of academic outputs and timely dissemination of performance reports.

The recommendations of this study directly respond to the identified challenges. First, the inconsistent application of performance monitoring procedures calls for standardization across universities to reduce perceptions of bias. Second, the widespread dissatisfaction with irregular feedback justifies the establishment of regular, structured feedback mechanisms that are developmental rather than punitive. Third, concerns about limited involvement in setting performance criteria highlight the importance of participatory appraisal systems that give staff ownership of evaluation processes. Fourth, irregular training for supervisors underscores the need to strengthen monitoring capacity through workshops and mentoring. Finally, qualitative responses pointing to the inefficiency of manual systems justify the recommendation to leverage digital platforms for timely and transparent performance tracking.

Standardize performance monitoring procedures within two academic years, with each faculty required to adopt a unified appraisal template approved by the university senate.

Establish quarterly feedback forums where heads of departments meet with staff to discuss appraisal outcomes.

Adopt a digital monitoring dashboard (e.g., KIU-MIS or Moodle plugin) within 12 months to streamline reporting and transparency.

Integrate performance monitoring results into annual promotion and training plans to ensure accountability and continuous improvement.

### Ethical approval statement

This study received ethical approval from the **Research Ethics Committee of Kampala International University,** Uganda. The approval was granted on **September 6** ^**th**^ **2024,** with the reference number KIU-2024-292. The ethics committee approved the research protocol, participant recruitment procedures, and data protection measures and from the Uganda National Council for Science and Technology (UNCST) under national approval number **SS3145ES**. uncst.go.ug

This study received ethical approval from the Research Ethics Committee of Kampala International University (Reference: KIU-2024-292, approved on 6 September 2024). Additional approval was obtained from the Uganda National Council for Science and Technology (UNCST approval number: SS3145ES). Before data collection, participants were provided with detailed information about the study’s purpose, procedures, risks, and benefits. Written informed consent was obtained from all participants, covering both the questionnaire survey and the interviews. Participation was entirely voluntary, with the option to withdraw at any stage. Confidentiality was ensured by anonymizing responses and securely storing all data on password-protected devices.

### Informed consent statement

Prior to data collection, all participants were provided with detailed information about the purpose of the study, the procedures involved, their rights as participants, and any potential risks or benefits. Written informed consent was obtained from all participants before their involvement in the study. This consent covered both the questionnaire survey and the interview guide, ensuring that participants understood and agreed to participate voluntarily in either or both aspects of data collection.

## Data Availability

Open Science Framework: Establishing the Perception of Academic Staff on Performance Monitoring in Private Chartered Universities in Western Uganda, https://doi.org/10.17605/OSF.IO/TCRZG ( silaji, T. et al., 2025a). This project contains the following underlying data:
•
*Survey responses.xlsx* (Raw data collected from academic staff survey on performance monitoring and organizational structure). Data is available under the terms of the License: CC0 1.0 Universal. Open Science Framework: Establishing the Perception of Academic Staff on Performance Monitoring in Private Chartered Universities in Western Uganda, https://doi.org/10.17605/OSF.IO/TCRZG ( silaji, T. et al., 2025b). This project contains the following extended data:
•
*Questionnaire.pdf* (The full questionnaire used to collect survey data from participants). •
*Interview guide.pdf* ( *Interview transcripts.docx* (Qualitative data from Deans)). • All other key documents that supported this research study Extended data is available under the terms of the License: CC0 1.0 Universal.

## References

[ref37] Abdullahi S Musa A Bello R : Performance management practices in Nigerian universities: Implications for faculty performance. *J. Afr. High. Educ. Stud.* 2023;12(3):45–63.

[ref39] Adams J Wang X : Performance monitoring, academic stress, and burnout in private universities. *High. Educ. Rev.* 2020;52(4):601–620.

[ref40] Adekoya OD Ajagbe AM : Decentralized performance evaluation and academic staff effectiveness in Nigerian universities. *J. Educ. Manag. Leadersh.* 2021;13(2):45–59.

[ref41] Adhikari R Shrestha P : Institutional monitoring practices and sustainable quality education in South Asian universities: Implications for SDG 4.7. *Int. J. Educ. Dev.* 2023;9(1):112–130.

[ref35] Adyanga P Okello D Wekesa S : *Performance monitoring and staff productivity in East African universities: A comparative study.* Nairobi: East African Academic Press;2022.

[ref1] Agyemang CB Ofei SB : Employee work engagement and organizational commitment: A comparative study of public and private sector organizations in Ghana. *European Journal of Business and Management.* 2013;5(24):2222–2839.

[ref38] Ahmed H Gohar F : Organisational design and employee performance in African higher education contexts. *Int. J. Educ. Manag.* 2019;33(7):1501–1516.

[ref42] Alhassan MH Habib SA : Adaptive monitoring mechanisms and academic excellence in West African higher education institutions. *Afr. J. High. Educ. Policy.* 2024;18(1):77–95.

[ref25] Atwebembeire J Malunda P : Stakeholder’s perception on financing of private education in Uganda. *Ugandan Journal of Management and Public Policy Studies.* 2018;1(1):79–88.

[ref2] Atwebembeire J Tumwesigye G Ahimbisibwe A : Performance appraisal practices and performance of academic staff in private universities in Uganda: A case study of Bishop Stuart University. *Int. J. Manag. Commerce Innov.* 2018;6(2):1111–1121.

[ref3] Barasa JK Kaabwe SM : Fallacies in policy and strategies of skills training for the informal sector: Evidence from Uganda. *Int. J. Educ. Dev.* 2001;21(1):55–67. 10.1016/S0738-0593(00)00012-6

[ref26] Bell GH Bitton A Desai EV : Health facility management and primary health care performance in Uganda. *BMC Health Serv. Res.* 2018;18(1):275. 10.1186/s12913-022-07674-3 35232451 PMC8886189

[ref43] Chen M : *Qualitative data collection and analysis techniques for applied research.* Sage Publications;2020.

[ref27] Christine VH Wanyama S Burton B : Stakeholders, accountability and the theory-practice gap in developing nations’ corporate governance systems: Evidence from Uganda. *Corp. Gov.: Int. Rev.* 2013;13(1):18–38. 10.1108/14720701311302396

[ref4] Daka H : Appraisal systems and academic staff perception in Lusaka’s private universities. *Journal of African Higher Education Management.* 2024;12(1):34–50.

[ref33] Deci EL Ryan RM : *Intrinsic motivation and self-determination in human behavior.* New York: Springer;1985. 10.1007/978-1-4899-2271-7

[ref34] Deci EL Ryan RM : The “what” and “why” of goal pursuits: Human needs and the self-determination of behavior. *Psychological Inquiry.* 2000;11(4):227–268. 10.1207/S15327965PLI1104_01

[ref28] Goldberg LR : An alternative “description of personality”: The Big-Five factor structure. * J. Pers. Soc. Psychol.* 1990;59(6):1216–1229. 10.1037/0022-3514.59.6.1216 2283588

[ref5] Ismail AI Raza A : The impact of performance management system on employee performance: A case study of university lecturers in Pakistan. *J. Hum. Resour. Manag. Res.* 2019;2019:1–10. 10.5171/2019.835079

[ref6] Jansen B Smith R : Linking performance feedback with academic staff development: A mixed-methods study. *High. Educ. Res. Dev.* 2021;40(1):120–136. 10.1080/07294360.2020.1799942

[ref36] Kabwe M : *Organisational structures and academic staff outcomes in Zambian higher education institutions.* Lusaka: Zambian Institute of Education Research;2024.

[ref29] Kansiime G : Performance management of the academic staffs in Ugandan public and private universities (Doctoral dissertation, Rhodes University). *Rhodes University Institutional Repository.* 2023. Reference Source

[ref7] Kansiime M Singh S : Evaluating performance management systems in Ugandan universities: A comparative analysis of public and private institutions. *African Journal of Educational Management.* 2023;15(1):74–89.

[ref30] Karuhanga BN : Evaluating implementation of strategic performance management practices in universities in Uganda. *Meas. Bus. Excell.* 2015;19(2):42–56. 10.1108/MBE-06-2014-0017

[ref31] Kendra C : The Big Five personality traits. *Verywell Mind.* 2016. Reference Source

[ref8] Kim J Lee S : Academic staff perceptions of performance monitoring and its influence on satisfaction and productivity in South Korean universities. *J. High. Educ. Policy Manag.* 2018;40(3):245–262. 10.1080/1360080X.2018.1462435

[ref9] Mohammadi S Karupiah P : The influence of performance appraisal practices on academic staff performance in Malaysian private higher education institutions. *J. Educ. Soc. Res.* 2020;10(1):142–150. 10.36941/jesr-2020-0013

[ref10] Molefe G : Performance measurement model and academic staff: A survey at selected universities in South Africa. *SA J. Hum. Resour. Manag.* 2012;10(2):1–9. 10.4102/sajhrm.v10i2.326

[ref11] Mutabaruka G : Performance appraisal system and employee productivity in Rwanda’s private universities: A case of Kigali Independent University. *East African Journal of Education and Social Sciences.* 2022;3(1):74–81. 10.46606/eajess2022v03i01.0148

[ref12] Nganyi EW Shigogodi JJ Owano AA : Enhancing performance management in public universities in Kenya through effective information communication technology. *International Journal of Academic Research in Business and Social Sciences.* 2014;4(9):266–278. 10.6007/IJARBSS/v4-i9/1176

[ref13] Nguyen TH Pham HL : Performance monitoring and academic engagement in Vietnamese higher education institutions. *Asian Educ. Dev. Stud.* 2020;9(4):553–570. 10.1108/AEDS-06-2020-0148

[ref14] Osei C Ackah D : Employee perception of performance appraisal system: A case study. *Int. J. Economics Commerce Manag.* 2015;3(1):1–11.

[ref15] Rwothumio JM Aguti JN Nkata JL : Performance appraisal and academic staff productivity in Ugandan public universities: A case study of Kyambogo University. *International Journal of Research and Innovation in Social Science (IJRISS).* 2021;5(2):2454–6186. Reference Source

[ref16] Sabi HM Uzoka F-ME Mlay SV : Cloud computing applications in the education sector: A review of adoption in developing countries. *Educ. Inf. Technol.* 2018;23(2):877–903. 10.1007/s10639-017-9633-4

[ref17] Silaji T Bagiwa ZL Muhammad T : Establishing the Perception of Academic Staff on Performance Monitoring in Private Chartered Universities in Western Uganda. 2025a, July 10. 10.17605/OSF.IO/TCRZG PMC12640481 41287836

[ref18] Silaji T Bagiwa ZL Muhammad T : Establishing the Perception of Academic Staff on Performance Monitoring in Private Chartered Universities in Western Uganda. 2025b, July 23. 10.17605/OSF.IO/TCRZG PMC12640481 41287836

[ref32] Ssemwanga SL Muyinda H : Emotional intelligence competence and job performance of academic staff in private universities in Uganda. *Int. J. Soc. Sci. Humanit. Res.* 2021;9(1):146–153.

[ref19] Thompson L Houghton JD : Performance monitoring perceptions and motivation among academic staff: A Self-Determination Theory perspective. *Educ. Manag. Adm. Lead.* 2019;47(2):292–309. 10.1177/1741143217745886

[ref20] Tibarimbasa AK : *Factors affecting the management of private universities in Uganda.* Makerere University;2010. (Doctoral dissertation). Reference Source

[ref21] Turk M Killumets E : Performance management of academic staff in Estonian universities: Problems and perspectives. *High Educ. Pol.* 2014;27:441–457. 10.1057/hep.2014.20

[ref22] Waweru NM Kalani VM : Commercial banking crises in Kenya: Causes and remedies. *Global Journal of Finance and Banking Issues.* 2009;3(3):23–29.

[ref23] Yousefi R Ahmad J Abdul Razak N : Performance appraisal and job satisfaction among academic staff in Malaysian private universities. *International Journal of Academic Research in Business and Social Sciences.* 2020;10(1):290–306. 10.6007/IJARBSS/v10-i1/6863

[ref24] Zhang Y Wang L : Equity-based performance monitoring and academic staff outcomes in Chinese universities. *Int. J. Educ. Manag.* 2022;36(6):1152–1170. 10.1108/IJEM-09-2021-0395

