# Supplementary material for: Establishing the Perception of Academic Staff on Performance Monitoring in Private Chartered Universities in Western Uganda
Source: F1000Res. 2025 Nov 26;14:805. Originally published 2025 Aug 20. [Version 3] doi: 10.12688/f1000research.167786.3 (PMC12640481; doi:10.12688/f1000research.167786.3)
Supplement: Peer review history

# Reviewer report: Reviewer response for version 3

**DOI:** 10.5256/f1000research.191468.r436100

**Published:** 2025-12-02

**Authors:** Samra Maqbool

**Affiliations:** 1 Beijing Normal University, Beijing, Beijing, China

The paper has been revised in accordance with the reviewer’s comments, and I recommend its acceptance.

Is the work clearly and accurately presented and does it cite the current literature?

Yes

If applicable, is the statistical analysis and its interpretation appropriate?

Yes

Are all the source data underlying the results available to ensure full reproducibility?

Yes

Is the study design appropriate and is the work technically sound?

Yes

Are the conclusions drawn adequately supported by the results?

Yes

Are sufficient details of methods and analysis provided to allow replication by others?

Yes

Reviewer Expertise:

Education

I confirm that I have read this submission and believe that I have an appropriate level of expertise to confirm that it is of an acceptable scientific standard.

# Reviewer report: Reviewer response for version 2

**DOI:** 10.5256/f1000research.187657.r426765

**Published:** 2025-11-22

**Authors:** Maulana Amirul Adha

**Affiliations:** 1 Universitas Negeri Jakarta, East Jakarta, Special Capital Region of Jakarta, Indonesia

1. Although the topic of performance monitoring in private universities in Uganda is highly relevant, the manuscript does not clearly identify the research gaps that differentiate this study from previous studies. The theoretical contribution is limited to rehashing concepts in the literature, rather than developing new perspectives on the empirical findings.

 2. Several sections appear repetitive, particularly in the literature review and discussion, where the same sources are cited repeatedly without providing additional analytical value.

 3. The quantitative results are presented only in tables, correlation values, and regressions without any explanation that would enrich the context of the findings. On the qualitative side, the researcher only presents a summary of informant comments without a structured thematic analysis. For example, there is no grouping of main themes, subthemes, or examples of quotations showing how the data were analyzed. Furthermore, the mixed-methods approach is not presented in an integrative manner; quantitative and qualitative results are presented side by side without linking them to strengthen the meaning of the research findings.

 4. The conclusions and recommendations often go beyond empirical evidence. For example, the proposed use of digital platforms is not strongly supported by either qualitative or quantitative data. The author needs to demonstrate a direct link between the field findings and the proposed solutions to make the recommendations more convincing and less normative.

Is the work clearly and accurately presented and does it cite the current literature?

Partly

If applicable, is the statistical analysis and its interpretation appropriate?

Yes

Are all the source data underlying the results available to ensure full reproducibility?

Partly

Is the study design appropriate and is the work technically sound?

Partly

Are the conclusions drawn adequately supported by the results?

Partly

Are sufficient details of methods and analysis provided to allow replication by others?

Yes

Reviewer Expertise:

Educational management, educational leadership, educational supervision

I confirm that I have read this submission and believe that I have an appropriate level of expertise to confirm that it is of an acceptable scientific standard, however I have significant reservations, as outlined above.

# Response

**Published:** 2025-11-22

**Authors:** Turyamureeba silaji

**Affiliations:** Foundations, Kampala International University - Western Campus, Bushenyi, Western Region, Uganda

**Response to Reviewer Comments**

 Dear Peer reviewer, We appreciate the thorough and constructive feedback provided to improve our Manuscript. Each comment has significantly strengthened the manuscript. Below, we present a point-by-point response and indicate the specific revisions made in the revised version.

**Reviewer Comment 1**

*“Although the topic of performance monitoring in private universities in Uganda is highly relevant, the manuscript does not clearly identify the research gaps that differentiate this study from previous studies. The theoretical contribution is limited to rehashing concepts in the literature, rather than developing new perspectives on the empirical findings.”*

**Response:**

 Thank you for this insightful comment. We have now clearly articulated the specific research gap within the Introduction section. The revised version highlights the limited empirical work on perceptual and motivational aspects of performance monitoring in Ugandan private chartered universities, distinguishing this study from prior work focused either on public universities or general performance management.

 We also strengthened the theoretical contribution by explicitly linking empirical results to the mechanisms proposed in Self-Determination Theory (SDT) and Expectancy Theory.

**Where Correction Was Made:**

 Introduction → Research Gap (new subsection added)

 Strengthened theoretical articulation in the last paragraph of the Introduction

 Improved integration of theory within the Discussion

**Reviewer Comment 2**

*“Several sections appear repetitive, particularly in the literature review and discussion, where the same sources are cited repeatedly without providing additional analytical value.”*

**Response:**

 We appreciate this observation. We revised the Literature Review to remove redundancy and integrated overlapping ideas into concise, analytical paragraphs. Instead of repeating the same studies, the revised section synthesizes literature to highlight consistent patterns, methodological gaps, and contextual differences relevant to private universities in Uganda.

**Where Correction Was Made:**

 Literature Review → Redrafted to remove repetition

 Discussion → Redundant citations removed; analytical synthesis strengthened

**Reviewer Comment 3**

*“The quantitative results are presented only in tables, correlation values, and regressions without any explanation that would enrich the context of the findings. On the qualitative side, the researcher only presents a summary of informant comments without a structured thematic analysis. There is no grouping of main themes, subthemes, or quotations showing how the data were analyzed. Furthermore, the mixed-methods approach is not presented in an integrative manner.”*

**Response:**

 We agree fully with this important comment. The revised manuscript includes:

 Detailed narrative explanations accompanying each quantitative table to contextualize patterns and statistical relationships.

 A rigorous thematic analysis for the qualitative component, including four main themes, sub-themes, and representative quotations from deans.

 A new Mixed-Methods Integration subsection showing convergence and complementarity between quantitative and qualitative findings (e.g., how qualitative concerns about inconsistency explain the moderate regression results).

 These revisions ensure methodological rigor and enhance the interpretive depth of the study.

**Where Correction Was Made:**

 Results → Quantitative narrative explanations added

 Qualitative Findings → Full thematic structure added with quotes

 Mixed-Methods Integration → New subsection added at the end of Results

**Reviewer Comment 4**

*“The conclusions and recommendations often go beyond empirical evidence. For example, the proposed use of digital platforms is not strongly supported by either qualitative or quantitative data. The author needs to demonstrate a direct link between the field findings and the proposed solutions to make the recommendations more convincing and less normative.”*

**Response:**

 Thank you for highlighting this issue. We revised the Conclusions and Recommendations to ensure that each is grounded strictly in empirical findings. Specifically, the recommendation for digital platforms is now supported by qualitative evidence referring to administrative delays and manual tracking challenges reported by deans. Normative statements have been removed, and all recommendations now align directly with the study’s data.

**Where Correction Was Made:**

 Conclusion → Revised to align with empirical findings

 Recommendations → Rewritten to directly reflect qualitative and quantitative results

 Added explicit justification for digital platforms based on documented implementation challenges

**Final Note**

 We thank the reviewer for providing constructive feedback that has substantially strengthened the manuscript. All suggested changes have been incorporated thoughtfully and thoroughly.

# Reviewer report: Reviewer response for version 2

**DOI:** 10.5256/f1000research.187657.r426772

**Published:** 2025-11-13

**Authors:** Samra Maqbool

**Affiliations:** 1 Beijing Normal University, Beijing, Beijing, China

The paper presents valuable insights into academic staff perceptions of performance monitoring in private chartered universities in Western Uganda. However, several major revisions are required:

1. The theoretical framework, especially Self-Determination Theory and Expectancy Theory, should be more explicitly tied to the research questions and analysis. Currently, it feels tangential. Ensure that the link between theory and empirical findings is more apparent.
2. While the mixed-methods design is appropriate, the paper would benefit from a clearer justification of the choice of sample size, particularly in the qualitative strand. Justify the selection of Deans and their relevance to the study.
3. The results section can be more concise. Avoid redundancy in presenting findings and make stronger connections between quantitative and qualitative results.
4. Recommendations should be more specific and actionable, including potential timelines or examples of how to implement improvements in feedback mechanisms.
5. Expand the literature review to provide a more comprehensive context for the Ugandan higher education system and how performance monitoring fits within broader regional trends.

Is the work clearly and accurately presented and does it cite the current literature?

Yes

If applicable, is the statistical analysis and its interpretation appropriate?

Yes

Are all the source data underlying the results available to ensure full reproducibility?

Yes

Is the study design appropriate and is the work technically sound?

Yes

Are the conclusions drawn adequately supported by the results?

Yes

Are sufficient details of methods and analysis provided to allow replication by others?

Yes

Reviewer Expertise:

Education

I confirm that I have read this submission and believe that I have an appropriate level of expertise to confirm that it is of an acceptable scientific standard, however I have significant reservations, as outlined above.

# Response

**Published:** 2025-11-13

**Authors:** Turyamureeba silaji

**Affiliations:** Foundations, Kampala International University - Western Campus, Bushenyi, Western Region, Uganda

**Response Letter to Peer Reviewer**

**Manuscript Title:** ***Establishing the Perception of Academic Staff on Performance Monitoring in Private Chartered Universities in Western Uganda***

**Authors:** Silaji T., Bagiwa Z. L., & Muhammad T.

**Manuscript ID:** F1000Res164151

**Date:** 13 ^th ^Nov, 2025

**Dear Reviewer,**

 We sincerely thank you for your thoughtful and constructive comments on our manuscript. Your feedback has been instrumental in improving the conceptual clarity, methodological justification, and overall coherence of the paper. Below are our detailed responses to each comment and the corresponding revisions made.

**1. Theoretical Framework (Self-Determination Theory and Expectancy Theory)**

**Reviewer’s Comment:**

 The theoretical framework, especially Self-Determination Theory and Expectancy Theory, should be more explicitly tied to the research questions and analysis. Currently, it feels tangential. Ensure that the link between theory and empirical findings is more apparent.

**Response:**

 We agree with this valuable observation. The theoretical framework section has been expanded to explicitly connect both Self-Determination Theory (SDT) and Expectancy Theory to the study’s objectives, variables, and empirical findings.

 Specifically, SDT has been linked to the need for autonomy and intrinsic motivation among academic staff within mechanistic organisational structures.

 Expectancy Theory has been related to how staff perceptions of fairness in performance monitoring influence effort, performance, and rewards. In addition, theoretical implications were incorporated into the *Discussion* and *Conclusion* sections to demonstrate how these theories explain the observed relationships between structure, monitoring, and staff performance.

**Location in Manuscript:** Pages 6–8 and 27–29.

**2. Mixed-Methods Design and Justification of Sample Size**

**Reviewer’s Comment:**

 While the mixed-methods design is appropriate, the paper would benefit from a clearer justification of the choice of sample size, particularly in the qualitative strand. Justify the selection of Deans and their relevance to the study.

**Response:**

 We have added a stronger rationale for both the quantitative and qualitative sample sizes. The quantitative sample of 386 academic staff was justified using the Krejcie and Morgan (1970) formula to ensure representativeness across faculties and academic ranks. For the qualitative strand, 10 Deans of Faculties were purposively selected because of their strategic administrative and supervisory roles, which provide deep insight into organisational structures, decision-making processes, and performance monitoring practices.Location in Manuscript: Pages 9–10 (Research Design and Sampling).

**3. Results Section – Redundancy and Integration**

**Reviewer’s Comment:**

 The results section can be more concise. Avoid redundancy in presenting findings and make stronger connections between quantitative and qualitative results.

**Response:**

 We have revised the *Results* and *Discussion* sections to eliminate repeated points and enhance integration. Each objective now begins with quantitative findings, followed by supporting qualitative evidence and interpretive links to literature. This structure ensures that the results are succinct and cohesive. The integration now clearly demonstrates how qualitative themes explain or extend the statistical results. **Location in Manuscript:** Pages 13–25.

**4. Recommendations – Specificity and Actionability**

**Reviewer’s Comment:**

 Recommendations should be more specific and actionable, including potential timelines or examples of how to implement improvements in feedback mechanisms.

**Response:**

 We have rewritten the *Recommendations* section to make it more practical and actionable. The new version includes specific strategies for implementing structured performance feedback systems, capacity-building initiatives, and gradual decentralisation of decision-making. Short- and medium-term implementation timelines (1–3 years) were also included to guide institutional planning and policy development.

**Location in Manuscript:** Pages 31–33.

**5. Literature Review – Contextual Expansion**

**Reviewer’s Comment:**

 Expand the literature review to provide a more comprehensive context for the Ugandan higher education system and how performance monitoring fits within broader regional trends.

**Response:**

 The *Literature Review* has been expanded to include a deeper contextual discussion of the Ugandan and East African higher education landscape. We integrated recent studies from Uganda, Kenya, Nigeria, and Zambia to illustrate how performance monitoring practices and organisational structures vary regionally. Additional references from Adyanga et al. (2022), Kabwe (2024), Abdullahi et al. (2023), and Ahmed & Gohar (2019) have been incorporated to strengthen the regional and comparative context. Location in Manuscript: Pages 5–7.

**Summary of Major Revisions**

- Expanded theoretical framework linking SDT and Expectancy Theory to findings.
- Justified sample sizes and selection of Deans for qualitative inquiry.
- Condensed and integrated results and discussion.
- Enhanced recommendations with actionable strategies and timelines.
- Broadened literature review to reflect regional higher education contexts.

We appreciate the reviewer’s insightful suggestions, which have greatly improved the depth and clarity of our manuscript. We believe these revisions fully address the concerns raised and enhance the manuscript’s contribution to knowledge and practice.

 Thank you once again for your thoughtful review.

**Sincerely,**

**Turyamureeba** **Silaji** (Corresponding Author)

 On behalf of the co-authors

 My warm regards and appreciation to you

# Reviewer report: Reviewer response for version 1

**DOI:** 10.5256/f1000research.184929.r408367

**Published:** 2025-09-02

**Authors:** Denok Sunarsi

**Affiliations:** 1 Universitas Pamulang, Tangerang, Banten, Indonesia

The article presents relevant findings on performance monitoring in Ugandan private universities. However, to be scientifically sound, it must address methodological clarity (sampling procedure, response rate), improve theoretical integration, and present more robust qualitative evidence. Greater detail on instrument validity, ethical safeguards, and alignment between findings and recommendations is required.

Is the work clearly and accurately presented and does it cite the current literature?

Yes

If applicable, is the statistical analysis and its interpretation appropriate?

Partly

Are all the source data underlying the results available to ensure full reproducibility?

Yes

Is the study design appropriate and is the work technically sound?

Yes

Are the conclusions drawn adequately supported by the results?

Yes

Are sufficient details of methods and analysis provided to allow replication by others?

Yes

Reviewer Expertise:

Management

I confirm that I have read this submission and believe that I have an appropriate level of expertise to confirm that it is of an acceptable scientific standard.

# Response

**Published:** 2025-09-02

**Authors:** Turyamureeba silaji

**Affiliations:** Foundations, Kampala International University - Western Campus, Bushenyi, Western Region, Uganda

**Letter to Reviewer**

**Dear Reviewer,**

 We would like to sincerely thank you for your thorough review and constructive comments on our article, “Establishing the Perception of Academic Staff on Performance Monitoring in Private Chartered Universities in Western Uganda.” Your feedback has been invaluable in strengthening the methodological clarity, theoretical integration, and overall scientific rigor of our work.

**In response to your comments, we have made the following revisions:**

 1.Methodological Clarity (Sampling Procedure and Response Rate):

 We expanded the Methods section to provide a step-by-step explanation of the stratified random sampling process. We also clarified the response rate: 450 questionnaires were distributed and 386 returned, yielding an 85.8% response rate.

 2.Instrument Validity and Reliability:

 Additional detail has been provided on both the quantitative questionnaire and qualitative interview guide. For the quantitative strand, content validity was ensured through expert review, construct validity through factor analysis, and reliability confirmed with Cronbach’s alpha (0.79–0.86). For the qualitative strand, face and content validity were established through expert review and pilot testing, with reliability strengthened through member checking, triangulation, and use of a consistent protocol.

 3.Ethical Safeguards:

 We elaborated on the ethical processes, specifying approval numbers from both Kampala International University Research Ethics Committee (KIU-2024-292) and the Uganda National Council for Science and Technology (SS3145ES). Details on informed consent, confidentiality, and voluntary participation have been expanded.

 4.Theoretical Integration:

 We strengthened the link between our findings and the guiding frameworks of Expectancy Theory and Self-Determination Theory. For example, we show how fairness and feedback relate to expectancy-value mechanisms, and how autonomy, competence, and relatedness emerged in our qualitative findings.

 5.Qualitative Evidence:

 We enriched the qualitative strand by incorporating additional verbatim quotes from Deans to illustrate themes of irregular feedback, lack of transparency, and limited involvement in performance monitoring.

 6.Alignment Between Findings and Recommendations:

 The Conclusion and Recommendations sections were revised to ensure each recommendation directly stems from study findings. For example, staff concerns about limited feedback informed the recommendation to institutionalize structured feedback mechanisms.

 We believe these revisions fully address your concerns and have significantly improved the clarity, rigor, and contribution of the article. We are grateful for your guidance, which has enhanced both the theoretical depth and practical implications of our work.

 Thank you again for your insightful review.

 Yours faithfully,

 Turyamureeba Silaji

 silaji.turyamureeba@studwc.kiu.ac.ug

 On behalf of all authors
